# DNA-Based Nanomaterials as Drug Delivery Platforms for Increasing the Effect of Drugs in Tumors

**DOI:** 10.3390/cancers15072151

**Published:** 2023-04-05

**Authors:** Anastasiya N. Shishparenok, Vitalina V. Furman, Dmitry D. Zhdanov

**Affiliations:** 1 Laboratory of Medical Biotechnology, Institute of Biomedical Chemistry, Pogodinskaya St. 10/8, 119121 Moscow, Russia; 2 Center of Chemical Engineering, ITMO University, Kronverkskiy Prospekt 49A, 197101 St. Petersburg, Russia; 3 Department of Biochemistry, Peoples’ Friendship University of Russia (RUDN University), Miklukho-Maklaya St. 6, 117198 Moscow, Russia

**Keywords:** DNA-based nanomaterials, tetrahedron, origami, nanotube, aptamer, drug delivery, endocytosis

## Abstract

**Simple Summary:**

The use of drugs based on nucleic acids is a promising direction in antitumor therapy. Some modified oligonucleotide analogs, such as antisense oligonucleotides, have been developed and used as innovative therapeutic agents in some areas of medicine. Many ways to build DNA nanomaterials with predefined shape and function characteristics have been designed. Thus, molecules of potent antitumor drugs, including doxorubicin, therapeutic oligonucleotides, and complex nanoparticles, have been loaded into or conjugated with DNA-based nanomaterials. It was found that DNA-based nanomaterials can increase the efficiency of drug uptake by cells. In this review, we would like to draw attention to some DNA-based nanomaterials, such as tetrahedrons, origami, DNA nanotubes, and aptamers, that have been used as carriers, drugs or target molecules for anticancer drug delivery.

**Abstract:**

DNA nanotechnology has significantly advanced and might be used in biomedical applications, drug delivery, and cancer treatment during the past few decades. DNA nanomaterials are widely used in biomedical research involving biosensing, bioimaging, and drug delivery since they are remarkably addressable and biocompatible. Gradually, modified nucleic acids have begun to be employed to construct multifunctional DNA nanostructures with a variety of architectural designs. Aptamers are single-stranded nucleic acids (both DNAs and RNAs) capable of self-pairing to acquire secondary structure and of specifically binding with the target. Diagnosis and tumor therapy are prospective fields in which aptamers can be applied. Many DNA nanomaterials with three-dimensional structures have been studied as drug delivery systems for different anticancer medications or gene therapy agents. Different chemical alterations can be employed to construct a wide range of modified DNA nanostructures. Chemically altered DNA-based nanomaterials are useful for drug delivery because of their improved stability and inclusion of functional groups. In this work, the most common oligonucleotide nanomaterials were reviewed as modern drug delivery systems in tumor cells.

## 1. Introduction

DNA nanostructures were initially introduced by Nadrian Seeman in the 1980s. Numerous unique nanostructures have been created by designing various techniques and modifications. With the discovery of DNA tetrahedra based on base pairing and sticky ends, periodic lattices consisting of mosaic assembly based on DNA origami and DNA building blocks were developed [1].

The development of DNA-based materials has gone from applying natural DNA structures to artificially assembled DNAs, such as aptamers, origami, tetrahedra, nanotubes, hydrogels, dendrimers, and various DNA-based nanoparticles [2]. These materials are biocompatible, accessible, structurally diverse, almost noncytotoxic, and have the possibility to be captured by cells without transfection, so DNA-based materials are more suitable than other nanodrug delivery systems [3,4,5]. Their nucleotide-based frames create a ground for editing and modifying DNA-based nanostructures. Ligands with different functions can be embedded into their backbones in a variety of ways: conjugating, intercalating, encapsulating, and loading [6,7]. As a result, multiple DNA-based assemblies have been developed and employed in various biological disciplines, particularly in drug delivery [8].

On the other hand, only several active targeting nanodrugs have been approved for clinical use [9]. Unpredictable targeting efficacy, which frequently differs from individual to individual due to the variability in receptor expression, is one of the main problems [10]. Another is that macromolecular ligands (such as epidermal growth factor receptor, low-density lipoprotein, transferrin, and others) have high immunogenic activity and steric hindrance [11]. Therefore, the creation and use of DNA nanomaterials in composition with various micelles, inorganic, and organic nanoparticles conjugated with molecules for active targeting, which allow overcoming these limitations, have been increasingly studied. This strategy has exclusive properties and promising capacities for improving drug delivery, lowering drug toxicity, and targeted drug administration [12,13].

This review summarizes the structure, assembly, types of modifications, biological applications and future perspectives for DNA nanomaterials such as tetrahedra, origami, nanotubes, and aptamers for drug delivery in cancer treatment.

## 2. DNA-Based Materials

### 2.1. DNA Tetrahedron

Since the one-step synthesis of a tetrahedron (TDN) was first introduced, it has become one of the most widely used DNA nanostructures in biomedicine. TDNs, like other DNA nanostructures, are formed by mixing all four DNA oligonucleotides during a thermal annealing process [14]. Since TDN can be modified with various agents and has good biocompatibility, the use of TDN can increase the effectiveness of off-target anticancer drugs [15]. According to Fan et al., TDNs may successfully enter the dermis layer of mice and people’s skin and load and distribute the drug doxorubicin (DOX) to subcutaneous tumor locations [16].

**Figure 1:**
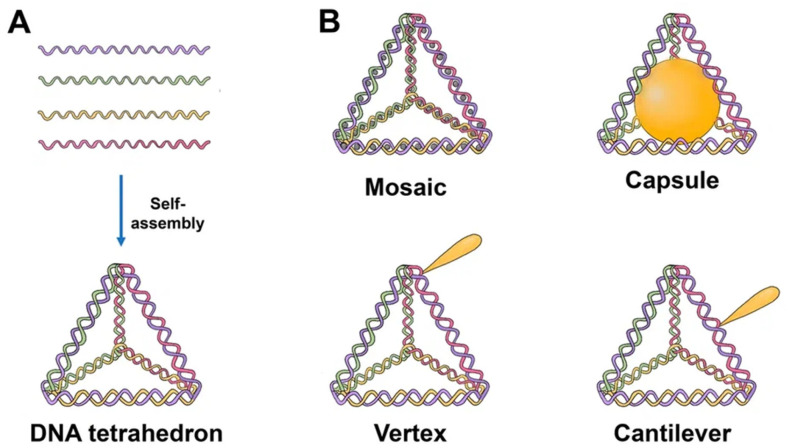
Schematics of the TDN structure and possibilities for drug loading. (**A**) Self-assembly of TDN by annealing. Each single strand consists of three sequence blocks, each complimentary to the sequence of another single strand. Hence, four triangles of DNA helices form a solid tetrahedral structure upon hybridization. (**B**) Tetrahedron modified with drugs. Mosaic: chemotherapy drug (doxorubicin, paclitaxel, actinomycin D). Capsule: nanoparticle (AuNP, for example), cytochrome c, peptide (melittin). Vertex: CpG, aptamer, mAbs, 5-fluorouracil, peptide. Cantilever: folate, siRNA, KillerRed, camptothecin.

The majority of TDNs used today are duplexes and double bundles, duplexes receiving the most research attention. TDNs can be modified with fluorescent dyes, bioligans, proteins, chemotherapy drugs, and various types of nucleic acids. Based on the various positions of the added functional groups or molecules, there are four major ways to modify TDN: vertex modification, mosaic modification, capsule modification, and cantilever modification (Figure 1) [17].

Vertex modification is the modification of functional groups in the top position of the TDN, such as azide groups for the ensuing click reaction or amino or sulfhydryl groups to stabilize the structure. Mosaic modifications occur when functionalized molecules or groups, such as chemotherapy drugs or fluorescent molecules, are used. The anticancer medicine is loaded into a DNA tetrahedron, which may then pass through the negatively charged cell membrane with minimal cytotoxicity, hence enhancing drug delivery. This method can also considerably lessen the drug’s negative side effects on the body.

The molecules of interest are put into the TDN in the case of capsular modification. By encapsulating nanoparticles in DNA, Mao et al. created nanocomplexes of a DNA tetrahedron with a gold nanoparticle. Cantilever modification of the TDN refers to the attachment of functional molecules or groups to the sides of the TDN. An example of such a modification was created by Tian et al. TDN associated with the angiopep-2 peptide [17].

Tetrahedrons have been used in a number of investigations thus far on medication delivery. TDNs created by Xie et al. and loaded with paclitaxel demonstrated stronger cytotoxicity on non-small cell lung cancer A549 cells and a PTX-resistant cell line than PTX alone. Moreover, drug resistance was overcome. TDNs appear to act as a P-glycoprotein inhibitor because the expression of the mdr1 gene and P-glycoprotein was shown to be downregulated in A549/T cells. Additionally, it was demonstrated that TDN-loaded PTX can induce apoptosis in A549/T cells [18].

Kim et al. showed that TDN can be employed as a carrier to deliver a variety of physiologically active molecules. [19,20]. They can include DNA-intercalating medicines such as doxorubicin, which can be easily loaded and efficiently delivered even to drug-resistant cells to demonstrate the desired action [21]. Photosensitizers may be loaded into TDN and transmitted into the cell with a high uptake efficiency since it is also known that methylene blue interacts with DNA duplexes [22,23].

Moreover, Kim et al. created a TDN streptavidin-mirror hybrid as an enzyme delivery vehicle. The biotinylated enzymes—caspase-3, Cre recombinase, and β-galactosidase—were loaded onto streptavidin. These TDNs were capable of penetrating cells, localizing in tumors, and delivering all of the mentioned enzymes, even intracellularly. As the hybrid could intracellularly carry the enzyme β-galactosidase, which is considerably larger than the antibody, it was concluded that the use of such a hybrid is not limited by the size of the delivery molecules [24].

The novel TDN was created by Tian et al. as a biocompatible nanocarrier of metal complexes. The TDN was linked noncovalently with two aptamers, AS1411 and MUC-1, and loaded with an iridium-based photocatalyst. The combined targeting of MUC-1 and AS1411 aptamers improved the selective cellular uptake and cytotoxicity of the iridium photocatalyst against U251 glioma cells. This compound successfully blocked signaling pathways by causing considerable fragmentation of mitochondria, inducing ROS-dependent apoptosis, and effectively inhibiting the migration of cancer cells [25].

Yang et al. integrated antisense oligonucleotides suppressing the c-raf protooncogene and nuclear targeting peptides into the double-bundle TDN. With the use of this delivery technique, the c-raf gene was successfully knocked down while also increasing the degree of target mRNA suppression at low concentrations in the nucleus and cytoplasm [26].

The use of affibody-TDNs as nanocarriers opens up new possibilities for the transport of nucleoside anticancer drugs. Affibody molecules are small polypeptides that can bind a number of different target proteins. The affibody molecule was conjugated to one of the vertices of the tetrahedron, and the 5-fluorouracil nucleoside analog (FUdR) preparation was attached to the other three vertices. Affibody-TDN complexes have demonstrated high selectivity and inhibition both in vitro and in vivo in HER2-overexpressing breast tumors [27].

### 2.2. DNA Origami

**Figure 2:**
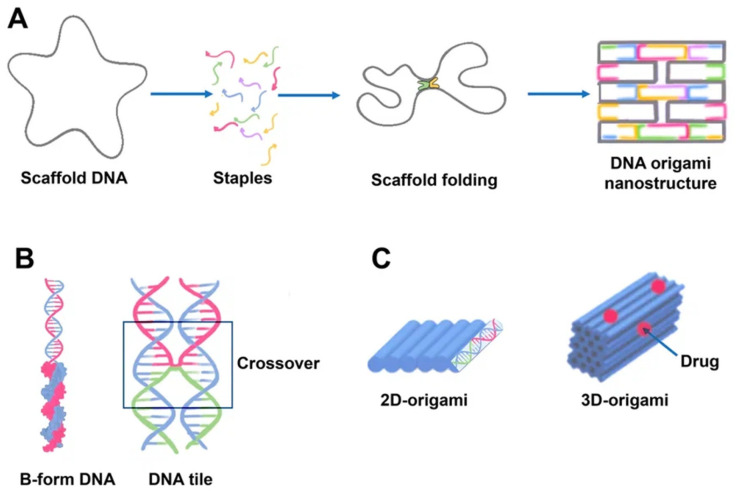
Schematics of DNA origami assembly and its structural features. (**A**) DNA origami assembly from the directed folding of the scaffold strand (gray) by using complementary staple strands (multicolored). Scaffold DNA functions as a guide or seed in the folding process, increasing folding efficiency and strand stoichiometry robustness. Each staple, for example, the blue strand, is incorporated specifically based on its sequence. The staples can connect distant sections of the scaffold by base pairing resulting in formation of a predefined form. The helices are formed by a scaffold chain (black) and a number of staple chains (colored). The lattice geometry is the consequence of crossings between the helix and its neighbors occurring seven base pairs apart. (**B**) The geometry of DNA crossovers in DNA helices for the creation of 2D origami designs. Every staple oligonucleotide binds to various regions of the scaffold DNA, producing double-helical tracts and connecting them. Individual interhelically connected tracts form a dense lattice in space. The connections, or crossovers, resemble an antiparallel Holliday four-way junction. Individual helical domains are connected by interhelix crossovers (in frame). Helices are linked to one another at regular intervals by junctions where scaffold or staples cross from one helix to the next. (**C**) 2D and 3D origami. DNA origami may be used to generate higher-order structures as a unit tile. A linear arrangement of DNA helices may be used to create 2D DNA origami. Cylinders are double-stranded DNA strands generated by base pairing of the DNA strands. 3D origami can be made by stacking multiple DNA helices on various lattices.

Paul Rotumend introduced the concept of DNA origami technology in 2006. DNA origami is a self-assembling nanostructure in which hundreds of short “staple” oligonucleotides fold a long single-stranded DNA “scaffold” into multilayer DNA assemblies [28]. The scaffold is folded into a predefined form using approximately 200 complimentary short strands (“staples”) by forming crossovers at every DNA helical turn. During thermal annealing, each staple specifically links various scaffold components together according to its own sequence, folding the scaffold into the desired form (Figure 2). The advantage of using DNA origami is concluded in the addressability of specific structural components with subnanometer accuracy and precision by changing individual staples [29].

DNA origami as combinations of single-layered complanate structures can be synthesized in different sizes (approximately 100 nm) and shapes (triangular, simple rectangular, five-point stars, complicated smiling faces) [8].

There are now a number of approaches for altering DNA origami structures. All of these techniques are based on the concept of backbone functionalization to obtain reactive end groups (e.g., biotinylation) of backbones and the addition of amino groups to backbones or chains associated with backbone extensions. These chains can be conjugated with other chains that carry drug molecules and nanoparticles [30].

**Figure 3:**
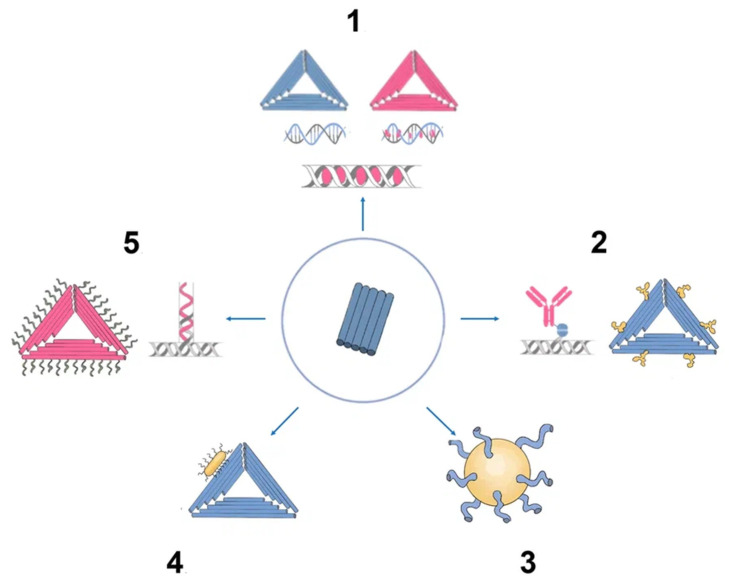
Origami as nanocarrier in cancer therapy. (**1**) Chemotherapy drugs. (**2**) DNA origami epitopes for IgGs. (**3**) DNA origami gold nanoparticle. (**4**) DNA origami gold nanorod nanocomplex. (**5**) Aptamer (e.g., MUC-1) + chemotherapy drug (e.g., doxorubicin).

There has been much interest in using DNA origami structures as drug delivery systems. First, DNA is a naturally occurring biomaterial that is both biodegradable and almost noncytotoxic. Second, various interactions (intercalation, base pairing, covalent binding) can easily load a variety of therapeutic compounds and materials onto carriers, including DOX, immunostimulatory nucleic acids, small interfering RNAs, antibodies, and enzymes (Figure 3). In addition, they can function as containers: DNA origami structures can house docking sites inside of them or in separate cavities that keep the payloads safe from the outside environment [31].

Recently, DNA origami has been used to develop useful cancer therapeutic applications, including sensory nanoplatforms and drug carriers [32]. When combined with anticancer medications, DNA origami-based molecular recognition parts can provide precise location data on tumor cells and treat cancer simultaneously [33].

Currently, research is being conducted to optimize the size and structure of DNA origami for passive targeting to tumor cells. Active targeting has been accomplished primarily by the incorporation of aptamers, the attachment of cell surface receptor ligands, and the use of cell-penetrating peptides. By hybridization, aptamer sequences can be easily integrated into origami backbones or conjugated on the origami surface [34].

Since extracellular and intracellular environments are chemically diverse, smart carriers must be capable of detecting environmental stimuli at different stages of delivery and switching their structures and properties to readjust. Douglas et al., for example, created a logic-gated nanorobot with a DNA origami container sealed by two aptamer motifs. This container opens only when both aptamers bind to the appropriate cell surface receptors, allowing conditional presentation of the drug molecules to certain cell types.

Jiang et al. discovered that when a 2D DNA origami triangle was compared to a 3D DNA origami tube structure, both structures were equally effective in delivering DOX inside cells of a breast cancer cell line. When compared to controls, both variants showed significantly higher cytotoxicity [35]. While drug DNA intercalation is a simple loading method, it does not provide any quantitative or qualitative control over the amount of loaded drug. The kinetics of drug release and the rate of carrier degradation in vivo are not strongly correlated, although the release of DOX from DNA origami has been examined in vitro [34]. Several articles have shown the therapeutic activity of different DOX-loaded DNA origami nanostructures employing in vitro and in vivo models. Using various DNA origami nanostructures, et al. demonstrated different efficiencies in DOX delivery to human breast cancer cells [36].

Jiang et al. noncovalently linked daunorubicin molecules to rod-like origami DNA nanostructures to overcome drug resistance in a leukemia cell line [35]. This rod-like origami DNA may be controllably loaded with daunorubicin, and this sort of origami has been demonstrated to dramatically boost medication effectiveness due to quick self-assembly and strong stability in cell culture, demonstrating a robust DNA nanostructure design. The scientists also demonstrated that at therapeutically relevant medication doses, origami DNA nanostructures might overcome drug resistance in leukemic cells [37].

BMEPC, a photosensitizer carbazole derivative created by Zhuang et al., was loaded into DNA origami. During irradiation, the DNA origami efficiently protected BMEPC molecules from photobleaching, and BMEPC fluoresced for a longer period of time; therefore, free radicals triggered cellular death in MCF-7 cells [38].

For drug delivery and medicinal applications, origami DNA nanostructures coupled to metal nanoparticles are becoming more widespread. A novel approach to cancer theranostics uses gold nanorods and DNA origami constructs. Compared to gold nanorods alone, DNA origami and gold nanorods together demonstrated improved cell uptake and greater antitumor effectiveness. This complex has the ability to image cells and photothermal ablation of malignant cells. In another example, the complex of nanoparticles with DNA origami provided a high ability to be loaded with ligands for binding to many molecules or drugs [39].

In addition, DNA origami has been created that can selectively target nucleolin in tumor blood vessels. This allowed the encapsulated thrombin to be exposed locally and promoted intravascular thrombosis, inhibiting tumor growth in mice and resulting in tumor necrosis [31].

### 2.3. DNA Nanotube

**Figure 4:**
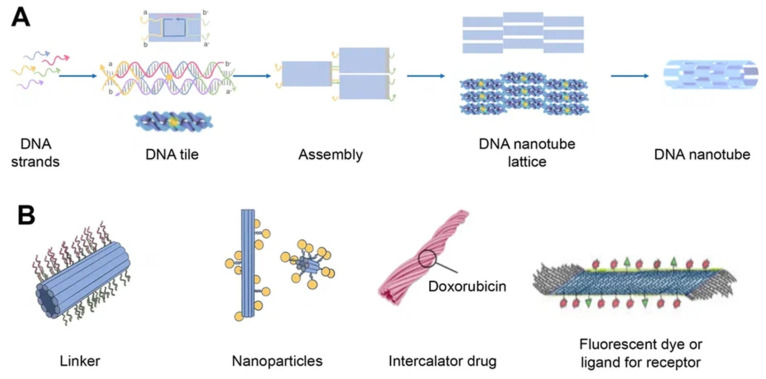
Schematics of DNA nanotube folding, modifications, and drug loading. (**A**) DNA nanotube self-assembly (schematically) using the single-stranded tile method. Five DNA strands form DNA double-crossover tiles. A DNA tile is formed with four short single-stranded sections called sticky ends (marked a, b, a′, and b′) that act as binding domains. Individual helical domains are connected by interhelix crossovers. Each domain is complementary to one domain of neighboring tiles and several domains hybridized with each other to form nanotubes (the single-stranded tile method of assembly of DNA nanotube). This interaction is provided due to complementary interactions of their sticky ends. Tiles can be schematically represented as molecular bricks with complementary connectors. The sticky end arrangement directs the hybridization of DNA tiles to form tubular DNA structures with a range of diameters. Their distribution is determined by the thermodynamics and kinetics of the DNA nanotube assembly process. (**B**) Modifications of DNA nanotubes: linker (CpG, cholesterol, aptamers, etc.); nanoparticles (Au); chemotherapy or photodynamic therapy drug; other ligands.

DNA nanotubes are crystalline self-assemblies made of 10 nm-diameter DNA tiles that can grow to tens of micrometers in length. The rigidity of DNA nanotubes is hundreds of times greater than that of double-stranded DNA in general [40]. DNA nanotubes are special 3D structures that have great promise for biomedical applications, such as filament supporting tracks and cargo transporting carriers [41]. Regardless of how they are assembled, DNA nanotubes can have well-defined or indefinite lengths. Single-stranded DNA tiles, multi-crossover tiles, DNA origami, and multi-rung design methodologies for DNA nanotubes are the key assembly techniques. Tile-based motifs have been employed as DNA nanostructure building blocks (Figure 4A) since the 1990s. Recently, single-stranded tile-based 2D and 3D DNA bricks were also developed. Self-assembly of DNA nanotubes is often achieved by heating and cooling mixtures of DNA strands with a given sequence, even though the complexity of DNA nanotubes varies. Importantly, the invention of 3D DNA nanostructure assembly helped the development and use of DNA nanotubes [42]. Typically, DNA nanotubes are created by vertically aligning DNA duplexes into a curved motif, then closing it, or by rolling and cyclizing a two-dimensional DNA origami array [43].

The transmembrane channels, bioreactors, and pharmaceuticals can be released over time in a precise nanoscale cavity that is provided by DNA nanotube channels. The exterior surface has a rigid scaffold and several organized connection locations that can be utilized as carriers and templates for cargo delivery. Biomimetic DNA nanotubes have shown considerable potential in bioimaging and therapies because of their strong biological compatibility and addressability [42].

DNA nanotubes can be modified with a range of ligands for various biomedical applications (Figure 4B). Among them, there may be DNA ligands, among which CpG and aptamers can be distinguished. Aptamers are usually used in this case for binding to a cellular target or as DNAzymes. In addition, intercalating drugs can be loaded into DNA nanotubes or various fluorescent dyes can be conjugated. DNA nanotubes can also be connected to liposomes or nanoparticles using single strand linkers [44].

DNA nanotubes are mostly able to absorb high concentrations of anticancer drugs. Unfortunately, the chemical mechanism of interaction between DNA nanotubes and medicines remains unclear. The impact of the structure of DNA nanotubes on drug dispersion and delivery, in particular, has not been investigated, limiting the utility of DNA nanotubes as drug carriers. Lijun Liang et al. used molecular dynamics simulations to investigate the potential of DNA nanotubes as drug delivery carriers. Due to electrostatic and van der Waals forces, certain hydrophobic anticancer medicines (doxorubicin, daunorubicin, Taxol, and vinblastine) might be stably absorbed at the ends of DNA nanotubes. Moreover, DNA nanotubes inhibited the aggregation of anticancer drugs in aqueous solutions. DNA nanotubes remain more stable after absorbing anticancer drugs [45].

DNA nanotubes were changed with simple ligands such as folic acid in the initial investigations. These DNA nanotubes have excellent cell membrane adherence and can quickly penetrate cancer cells. An increase in the quantity of folic acid fragments led to a 10% increase in DNA nanotube internalization. Several subsequent studies have implemented this change with variable degrees of success [46].

Another well-known modification of DNA nanotubes is the use of Cy3 fluorescent dye, which was delivered to KB epidermal nasopharyngeal carcinoma cells. Cy3 is known to have red fluorescence, which makes it easy to visualize. The combination of folic acid as a ligand for cancer cell receptors and Cy3 as a fluorescent imaging agent has been used to produce multifunctional nanotubes. The resulting DNA nanotubes could be efficiently absorbed by cancer cells without exhibiting cytotoxicity [47].

In addition, cholesterol-modified DNA nanotubes conjugated to cytochrome C have been used by Kokabey et al. for cancer cell apoptosis. The quantity of conjugated cholesterol molecules affects the efficiency with which DNA nanotubes bind to the plasma membrane. The death of cancer cells was linked to an increase in cell membrane permeability and only partially reliant on caspase activity, which in this circumstance suggests that cancer cells underwent both apoptosis and necrosis [48].

Li et al. created telomerase-responsive and nucleolin-targeted DNA nanotubes for drug delivery. Following the Förster resonance energy transfer (FRET) signal shift and RTA-induced cell death, the aptamer-functionalized DNA nanotubes loaded with RTA (ricin A chain) demonstrated improved tumor access and precise drug release in response to tumor cell telomerase. The DNA nanotubes were also effectively used in vivo, when after systemic injection, tumor growth in mice harboring xenografts was clearly inhibited [49].

### 2.4. Aptamers

Aptamers are typically peptides or single-stranded DNA or RNA that are short in sequence and can bind to cellular targets with high selectivity. Secondary or tertiary structures of aptamers facilitate target binding and determine specificity and affinity [50]. Aptamers can be employed for targeting nanocarriers toward tissue overexpressing the target antigens [51]. Similar to DNA, aptamers can combine to generate complementary base pairs, which can then be used to build a variety of complex structures. They can be secondary structures such as the kissing hairpin, stem, loop, bugle, and pseudoknot. These secondary structures can then be combined to create certain three-dimensional structures, which the cell’s target molecule can subsequently recognize. The major forces that result in the creation of a three-dimensional structure and the attachment of an aptamer to a target include hydrogen bonds, van der Waals forces, and hydrophobic and electrostatic interactions. Similar to how antibodies bind to antigens, the creation of aptamer-target complexes is driven by a unique 3D interaction [52].

Systematic evolution of ligands by exponential enrichment (SELEX) selects nucleic acid aptamers from a library of random sequences, which then bind to the selected compounds with great specificity and affinity. Exponentially enriching a population of random sequence nucleic acid libraries enables the SELEX technique to evolve and select molecules with the highest affinities. SELEX is used for nucleic acids because of the possibility of conveniently amplifying affinity-selected molecules by RT–PCR or PCR. Aptamers can circumvent some of the disadvantages associated with the use of antibodies. For instance, aptamers are produced in vitro and can be chosen to target almost any protein, including toxins or nonimmunogenic proteins, in a relatively short amount of time, whereas the use of living animals is a drawback for antibody production [53].

Aptamers have received much attention recently in the field of biomedicine. Aptamers have characteristics similar to those of antibodies as well as particular benefits such as thermal stability, simplicity in synthesis, reversibility of target binding, and minimal immunogenicity.

**Figure 5:**
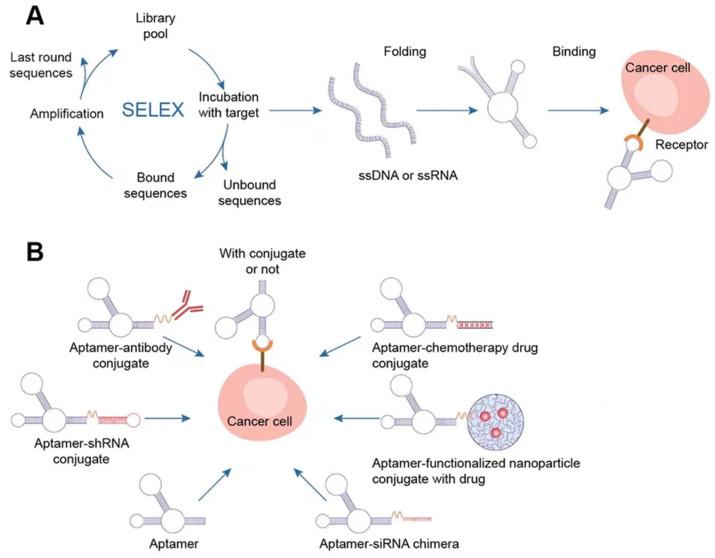
Aptamers. (**A**) Schematics of SELEX, aptamer folding, and binding to a target protein. A target-aptamer complex is created when the aptamer folds into a certain three-dimensional structure and interacts with a target molecule (such as a protein). (**B**) Aptamers for drug delivery in cancer.

Chemical techniques make it simple to change aptamers by adding functional groups and/or lengthening them (Figure 5) [50]. Changes can confer nuclease resistance and prolong the circulation half-life. For instance, aptamers’ circulation half-life can be extended to many hours by adding carrier molecules to their ends, such as polyethylene glycol or cholesterol [54]. Moreover, aptamers might be conjugated to therapeutic compounds such as medications, carriers for drugs, poisons, or photosensitizers [53].

There are several methods for the direct conjugation of aptamers to various secondary DNA structures, such as chemotherapy drugs or therapeutic oligonucleotides (siRNAs, miRNAs, and anti-miRNAs), that are easy to deliver and affordable. These methods take advantage of the chemical characteristics of aptamers. Drug compounds can simply be conjugated to aptamers via covalent or noncovalent bonding for targeted therapy. Most researchers have discussed the coupling of chemotherapeutic DNA structures to aptamers in their articles, and DOX is one of the most widely used drugs in this context [55]. Different DNA nanocarriers have been combined with therapeutic molecules that have unique properties. The most appealing nanostructures contain aptamers with chemotherapy drugs (DOX, PTX, 5-FU, etc.). They effectively damaged tumors, overcame multidrug resistance, and promoted photodynamic abilities. The idea of multifunctional complexes was brought up to improve their targeting capabilities. Moreover, complexes with aptamers play a significant role in immunostimulation, biosensing, and bioimaging when combined with fluorescent dyes (such as FAM) and bioactive DNA molecules (such as CpG) [8].

Some clinical investigations have been carried out to investigate the possibility of using aptamers in medicine. Pegaptanib, an RNA aptamer that targets VEGF in age-related macular degeneration, is the only aptamer that has been commercialized thus far. Nonetheless, more aptamers, such as Zimura, Fovista, NOX-H94, and BT200, are still being clinically tested [55].

Approximately 50 national clinical trials of aptamers are currently underway. Clinical trials using aptamers for the detection of cancer are also ongoing. In a clinical trial that was launched in 2015 (NCT02957370), novel bladder cancer biomarkers were found in urine samples. Ptamers may hold promise for in vivo tumor imaging or clinical diagnosis. Protein tyrosine kinase-7 (PTK7), which is expressed in a variety of human malignancies, has a particular ligand that has been discovered as a single-stranded DNA aptamer (Sgc8). The 68Ga-tagged aptamer was studied as a novel radiotracer for PTK7 positron emission tomography to distinguish between benign and malignant colorectal cancer in the most recent trial of this aptamer (NCT03385148).

Aptamers have been utilized to target cancer stem cells, produce laboratory tumor models, and create a novel breast cancer diagnostic system in another diagnostic clinical study (NCT01830244). A few fundamental studies are currently undergoing clinical trials, and several aptamers are offered commercially [56].

#### 2.4.1. AS1411 Aptamer

The human genome contains approximately 376,000 potential G-quadruplex sequences, including significant replication origins, telomeres, and gene promoter regions. G-quadruplexes in these locations regulate transcription, translation, and DNA replication as well as inhibit telomerase activity—these are key biological functions. It has been demonstrated that some artificially created G-quadruplex sequences are biologically active. The use of G-quadruplex structures is considered a promising direction for cancer treatment. The synthetic G-quadruplex AS1411 is employed to slow the growth of malignant tumors, leaving normal cells intact [57]. AS1411, a 26-mer DNA aptamer with a G-quadruplex structure known as a non-SELEX aptamer that binds to nucleolin, was discovered serendipitously by Bates et al. The molecular target of the AS1411 aptamer is nucleolin protein, which is found mainly in the nucleolus and distributed in the cytoplasm as well as on the cell surface.

Nucleolin is involved in a wide variety of cellular processes, including cell adhesion, cell division and migration, regulation of rRNA transcription, modification and processing of mRNA, regulation of telomerase maintenance, participation in DNA repair reactions, and cell growth. Nucleolin also plays a regulatory role in the maintenance of telomerase [58]. Nucleolin initiates and activates several signaling pathways, including TGF-, PI3K-AKT, epidermal growth factor-induced ERK, CXCR4, and CCR6 pathway signaling [59].

Researchers have widely employed the AS1411 aptamer as a therapeutic agent for numerous malignancies in vitro and in vivo, and it has also undergone clinical studies in humans. The therapeutic action of the AS1411 aptamer is most likely due to the degradation of the BCL-2 protein mRNA and the disruption of nuclear factor-kB signaling within cells [60].

A phase I clinical trial demonstrated that AS1411 has no significant toxicity in cancer patients and has high therapeutic potential. In addition, aptamers can be utilized as vectors by focusing on the markers of the cell surface to enter cancer cells with medicines, proteins, radionuclides, and nanoparticles [57]. AS1411 has been widely used to transport photosensitizers or chemotherapeutic drugs, but other aptamers have been reported infrequently in this area [61].

The Rosenberg team conducted a phase II clinical study that showed effectiveness against metastatic renal cell carcinoma in conjunction with cytarabine in patients with acute myeloid leukemia with minimum toxicity [62]. There are no clinical trials of AS1411 presently underway, and Advanced Cancer Therapies has acquired the rights to the AS1411 aptamer and renamed it ACT-GRO-777 [59].

Clinical trial data for AS1411 have demonstrated that the drug is well tolerated by patients and does not have any negative side effects that are life-threatening. Acute myeloid leukemia and renal cell carcinoma both had poor overall response rates, but at least seven individuals, three with renal cell carcinoma and four with AML, had long-lasting clinical responses in which their malignancy was eliminated or greatly shrank [63].

Although being taken out of clinical trials, AS1411′s structural optimization and the purpose of its nanomaterial remain important. Research into cancer therapy can be considerably advanced by the creation of AS1411 nanomaterial complexes to target molecules, for example, for the identification of cancer cells and in preventing tumor growth. AS1411 can combine with different nanoparticles, liposomes, and quantum dots and provide directed drug delivery to nucleolin-positive cells [64].

#### 2.4.2. MUC-1 Aptamer

MUC1 is a protein that is located on the surface of the majority of normal secretory epithelial cells. MUC1 can occur in two forms: underglycosylated (uMUC1) and tumor-associated (tMUC1) (TA-MUC1) [60]. The TA-MUC1 protein is abnormally overexpressed in many forms of cancer, including invasive lung cancer, pancreatic cancer, prostate cancer, ovarian cancer, primary lung cancer, and breast cancer, and occasionally in circulating tumor cells [65]. TA-MUC1 overexpression enhances tumor cell invasiveness, metastasis, cell proliferation, and chemoresistance [66].

The first MUC1 aptamer against the monoclonal antibody C595 was discovered by Missalidis et al. A number of MUC1 aptamers, including S1.1, S2.2, 5TR1, 5TRG2, MA3, and GalNAc3, that were chosen against various MUC1 receptor epitopes have also been identified [67].

The MUC1 aptamer was coupled with polimers or nanoparticles (NPs) for targeted drug delivery. The MUC1 aptamer’s circulatory half-life is lengthened when it is conjugated to poly(ethylene glycol) (PEG), which also imparts resistance to serum nuclease activity. For targeted administration of paclitaxel (PTX), the MUC1 aptamer S2.2 linked to poly(lactic-co-glycolic-acid) (PLGA) was employed (PTX). MUC1-conjugated nanoparticles loaded with PTX were substantially more harmful than empty NPs or NPs loaded with PTX, according to in vitro cytotoxicity experiments on MUC1-positive and MUC1-negative cell lines. Chitosan nanoparticles and the MUC1 aptamer were utilized by Sayari et al. to circumvent the hydrophobicity of the SN38 medication and lessen its negative effects. The resulting complex could be effectively internalized into MUC1-positive cells. Additionally, the MUC1 aptamer was conjugated to superparamagnetic iron oxide NPs and DOX, a DNA dendrimer, for the delivery of epirubicin [65]. Moreover, MUC1 aptamer-functionalized chitosan-coated human serum albumin NPs were used to create a selective drug carrier in MCF7 and T47D cell lines [68].

#### 2.4.3. PSMA Aptamer

PSMA, also known as glutamate carboxy-peptidase, is found in high concentrations on the surface of prostate cancer cells. According to estimates, prostate cancer cells express between 100,000 and 1,000,000 PSMA molecules per cell. [69]. PSMA can also internalize PSMA-bound ligands into cells [70].

Lupold and colleagues were the first to use an RNA aptamer to detect PSMA in a prostate tumor in 2002. Many anti-PSMA aptamers have been identified since then, including xPSM-A9, xPSM-A10, Aptamer A10, xPSM-A10 e3.2, xPSMA9 g, xPSMA-A9 L, and A10-3.2 [71].

Aptamers A10 and A9 are two commonly utilized aptamers in the targeting of prostate cancer cells. Its key benefit over competing anti-PSMA antibodies is their lower immunogenicity and excellent purity, which ensures nontoxicity and safety. PSMA aptamers are mainly applied as carriers to target drugs or simply to make them more bioavailable in prostate cancer [72].

Farokhzad et al. showed the usage of nanoparticle-aptamer bioconjugates in 2004 by conjugating the 5′-end of A10-3 to PLGA controlled-release NPs [73]. A10-3 conjugation greatly improved particle binding, uptake, and drug delivery to PSMA-positive prostate cancer cells and tumors in vitro and in vivo [73,74,75]. Applications of these aptamers have been successful with other nanoparticles, including quantum dots, superparamagnetic iron oxide nanoparticles, gold nanoparticles, and others [70].

There are some examples of aptamer–drug conjugates for prostate cancer in the literature. The first miRNA-aptamer conjugate was described in 2006 by McNamara et al. The authors developed RNA chimeras in which the A10 RNA aptamer was covalently linked to therapeutic miRNAs targeting PLK1 and BCL2 [76]. Dhar et al. found that nanoparticles with the A10 aptamer improved the in vivo pharmacokinetics of cisplatin, making the drug more tolerable and effective than cisplatin alone in a xenograft mouse model [74]. Docetaxel (DTX) is another drug that has also been utilized in aptamer technology. DTX-encapsulated PLGA-b-PEG NPs functionalized with A10 RNA aptamer improved targeted drug delivery and uptake [77]. The aptamer conjugated with nanoparticles and DTX was also found to have a greater anticancer impact in vivo in a tumor model using LNCaP cell xenografts and to be more efficient at inducing apoptosis of LNCaP cells than NPs without DTX in a study by Chen et al. [78].

PSMA aptamer was also used in complex with liposomes [79]. For example, unimolecular micelles in complex with aptamers have been used as vehicles for transporting DOX to prostate cancer cells. These conjugated molecules accumulated in cancer tissue more effectively than those lacking aptamers in their systems [80]. Aptamers conjugated with liposomes have been a popular research tool for prostate cancer therapy. Bandekar et al. investigated the ability of targeted liposomes filled with PSMA aptamer (Ac-225) to selectively kill PSMA-expressing cells performing targeted antivascular radiotherapy [81]. In addition, gold nanoparticles loaded with DOX and conjugated with aptamers were tested in LNCaP cells. It was found that such complexes can be used for both imaging and therapy of prostate tumors [82].

The FDA recently approved Lutetium-PSMA-617 radioligand therapy for the treatment of prostate cancer [83].

#### 2.4.4. CD28 Aptamer

Cluster of differentiation (CD) proteins are often used to create aptamers. Depending on the level of aptamer oligomerization, aptamers have the unusual ability to function both as CD receptor agonists and antagonists [84].

CD28 is one of the main costimulatory receptors responsible for the proper activation of T-lymphocytes. The first CD28 aptamers were isolated in 2013. As a monomer, one of them interfered with the binding of CD28 to its ligand B7, which is expressed on the surface of activated antigen-presenting cells, excluding the costimulatory signal, while the other was inactive. Upon dimerization of any of the anti-CD28s obtained, an artificial costimulatory signal was observed. Additionally, different agonistic structures were constructed for each anti-CD28 aptamer, one of which (CD28Apt7-dimer) demonstrated significantly improved costimulatory properties, surpassing the agonistic effect of the anti-CD28 antibody. The CD28 agonist aptamer has been shown to enhance the cellular immune response against idiotype lymphoma in vivo [85].

In the following studies of the authors, the novel bivalent MRP1-CD28 aptamer was developed using combinatorial peptide-Cell High Throughput SELEX technology. The identified anti-MRP1 aptamer was able to target chemotherapy-resistant tumors whose cells express the MRP1 protein. The previous authors showed that CD28 agonist aptamers could induce a potent proliferation signal on CD28 T-lymphocytes and promote an immune response against tumor antigens when used as vaccination adjuvants. Thus, a translational whole-cell vaccine (Aptvax) has been developed based on this MRP1-CD28 aptamer conjugate. For this purpose, a strategy was used to transform one’s own tumor in situ into an endogenous vaccine by irradiating melanoma tumor cells and coating them with CD28 costimulatory ligands. These aptamers enhance the costimulatory signal of CD28 to tumor-infiltrating lymphocytes. At the same time, in melanoma-bearing mice, which were injected with this bivalent aptamer, it was noted to cause a strong immune response, reduce tumor growth, and improve survival [86].

In another study, self-assembling multivalent CAR-like aptameric nanoparticles were created that can activate T cells by targeting mouse B16 melanoma tumor cells. It has been demonstrated for the first time that polyvalent aptamer nanoparticles can perform the function of CAR-T cells. CAR-like polyvalent aptamer nanoparticles (X-polymers) were assembled into a scaffold using a CD28 aptamer dimer (CD28Apt7), a CTLA-4 protein tetramer (associated with cytotoxic T-lymphocytes), and an RNA aptamer (Del60) and labeled with a folic acid fragment of ssDNA by a three-way junction scaffold. The results showed that X-polymers can affect both T cells and B16 cells in vitro. With anti-CD3 antibodies providing the first costimulatory signals, X-polymers can activate T cells, converting them into CAR-like T cells, which can inhibit melanoma growth in vitro by blocking CD28 and CTLA-4 [87].

CD28 aptamers have also been used in a study on specific inhibition of Treg function for antitumor immunotherapy. Treg cells possess the specific marker Foxp3. One of the Foxp3 inhibitors that can cross the membrane is the P60 peptide, which has low specificity and requires high doses. Therefore, the P60 Foxp3 peptide was conjugated to an aptamer targeting CD28 to deliver the peptide to cells expressing CD28. The AptCD28-P60 construct significantly increased the potency of the unconjugated P60 peptide. This approach has been used to inhibit Treg function and has shown a significant improvement in the induced immune response resulting in a reduction in tumor burden [88]. 

In another study, an aptamer nanodevice was created to dynamically modulate membrane receptor signaling and, thus, reduce cellular response. Combining reversible conformation switching with aptamer-based molecular recognition, this nanodevice has demonstrated superior efficacy in the dynamic regulation of T-cell immunity mediated by the CD28 receptor [89].

#### 2.4.5. CD44 Aptamer

CD44 is a cell surface glycoprotein that is involved in cell contacts, proliferation, and migration [72]. For the CD44 protein, it was shown that its CD44s and CD44v isoforms have different structures in cancer and normal cells. In this regard, some anti-CD44 aptamers have been isolated based on different structures of CD44 isoforms, which may be important in the development of biosensors for tumor differentiation from normal cells [90].

In 2022, several CD44 aptamers were reported in papers. The first is a DNA aptamer targeted at the extracellular N-terminal hyaluronan binding domain (HABD) of CD44. It has been shown to bind to several ovarian cancer cell lines with a KD of 181 nM. The second CD44 aptamer was a 2′-F-pyrimidine-modified RNA aptamer. The binding affinity of this CD44 RNA aptamer to recombinant CD44 protein was 81 nM, and it was capable of binding breast cancer cell lines. This RNA aptamer has also been demonstrated in vitro to induce apoptosis in various CD44-positive ovarian cancer cell lines and to decrease tumor spread in a mouse xenograft model. Another study found that gefitinib-loaded nanomicelles conjugated to this CD44 RNA aptamer prevented the development of spheroids in CD133-positive lung cancer cells.

The S5 rev aptamer, the third CD44 aptamer to be disclosed, likewise targets the recombinant HABD protein. This aptamer was cytotoxic to the NB4 leukemic cell line and had a KD of 238 nM. The fourth aptamer, CD44-Apt1, has dual targets and bears the designation CD44-Apt1. When conjugated with 5-FU, CD44-Apt1 demonstrated efficient uptake of 5-FU by HCC cells, which significantly increased the toxicity of the drug by more than a thousand times [91,92].

There are several studies on the use of the SD 44 aptamer conjugated with other aptamers. The CD44-anti-PD-L1 aptamer has been used in a targeted drug delivery system using nanosized liposomes. These liposomes have been loaded with loaded DOX and IDO1 siRNA and conjugated to CD44 and anti-PD-L1 DNA aptamers that target breast cancer cells and inhibit the PD-1/PD-L1 interaction between cancer cells and T cells [93]. An anti-EGFR-CD44 aptamer was used to target doxorubicin loaded in solid lipid nanoparticles to triple-negative breast cancer cells in which the receptor is overexpressed. Dexamethasone was chemically attached to the surface of the nanoparticles for more efficient delivery of doxorubicin to the nucleus of MDA-MB-468 cells. It was found that this designed nanoparticle was more effective than its components alone in inhibiting MDA-MB-468 cell proliferation [94].

In recent years, nanoparticles have been used to target CD44-overexpressing CSCs. In one study, a pentamer nanocomplex was synthesized to specifically target triple-negative breast cancer cells overexpressing CD44 receptors, including CSCs. A pentameric nanoparticle complex was designed and synthesized for targeted drug delivery of DOX to tumor cells overexpressing CD44. The novel five-component complex was designed Beals et al. It combines inorganic and polymeric nanoparticle techniques by incorporating CD44 DNA aptamer for cellular targeting and thiolated hyaluronic acid to stabilize Au NPs and improve drug loading. After 8 h, the in vitro release of DOX was found to be at its peak. In CD44+ cells compared to CD44 cells, the resultant complex was almost an order of magnitude more effective than DOX alone, and significantly decreased CSC self-renewal [95].

#### 2.4.6. CD71 Aptamer

CD71, also known as the transferrin receptor, is involved in cellular iron uptake and is overexpressed in rapidly proliferating cancer cells [96]. Several human malignancies, including cervical, breast, esophageal, pancreatic, and renal cancer, have been shown to have high CD71 expression [97].

An anti-CD71 DNA aptamer called XQ-2d was created by Wu et al. XQ-2d has a high affinity for pancreatic cancer cells. CD71 knockdown was found to abolish XQ-2d binding, and XQ-2d binding affinity was related to CD71 membrane-bound protein and not to total CD71 levels. The authors developed a targeted therapy for pancreatic cancer using a complex based on the XQ-2d aptamer and DOX [96].

Conjugation of the XQ-2d aptamer with monomethylauristatin E to target uveal melanoma cells was described by Zhang et al. In a mouse xenograft model, this complex was utilized to effectively target and inhibit the progression of cancer [98].

#### 2.4.7. CD117 Aptamer

Protein tyrosine kinase Kit (CD117), also called mast and stem cell growth factor receptor (SCFR), is a receptor tyrosine kinase derived from the KIT gene [99].

High levels of the transmembrane receptor CD117 are present on the leukemia cells of 95% of patients with relapsed acute myeloid leukemia (AML). Additionally, the survival prognosis for CD-117-positive AML patients was poorer than in situations where no expression was observed. Patients with high levels of CD117 expression had a lower rate of full remission [100].

To specifically target AML cells, a single-stranded DNA aptamer designed for CD117 has been created. Using a hybrid selection method, Zhao et al. discovered a single-stranded DNA aptamer sequence that is unique to CD117. The aptamer was then combined with methotrexate (MTX) to create an aptamer–drug complex. This complex selectively suppressed the growth of primary AML cells while having no effect on the patients’ normal bone marrow cells [101].

The CD117 aptamer has also been developed for targeted detection of gastrointestinal stromal tumors (GISTs). The KIT aptamer bound extracellular KIT in a manner similar to KIT staining with monoclonal antibodies. The KIT aptamer binds to dissociated primary human GIST cells regardless of mutations. The anti-KIT DNA aptamer was found to be highly specific for labeling GIST cells in vitro, intact human GIST tissue ex vivo, and peritoneal xenografts in mice [102]. Another study was looking for aptamers that can bind to the kinase domain of wild and mutant c-KIT proteins. One candidate, designated V15, might selectively inhibit the in vitro kinase activity of the c-KITD816V mutant with an IC50 value nine times greater than that of sunitinib among the aptamers produced by SELEX. H5/V36, a different aptamer, has shown the capacity to distinguish among c-KIT kinases [103].

#### 2.4.8. CD133 Aptamer

CD133 is a membrane glycoprotein that has been glycosylated. It is linked to the Notch signaling system, which regulates cell formation and influences their capacity to self-renew, proliferate, survive, differentiate, and undergo apoptosis [104]. In cancer research, CD133 is known as a cancer stem cell marker in glioblastomas [105].

Shigdar et al. isolated and characterized two RNA aptamers (A15 and B19) that selectively bind CD133. When compared to the same antibody in 3D cell culture, both aptamers exhibit high tumor penetration and accumulation [106].

CD133 is a reliable liver cancer stem cell marker. A specific RNA aptamer was developed against CD133, which was then loaded with the anticancer drug doxorubicin. It has been found that the CD133 aptamer can preferentially deliver doxorubicin to CD133-expressing liver cells with efficient drug accumulation and retention. Additionally, the CD133 aptamer blocked the self-renewal ability of liver cancer stem cells and weakened their stem phenotypes in vitro or in vivo. CD133-apt-DOX significantly inhibited the tumor growth of patient-derived organoids and reduced the growth of xenograft tumors in nude mice [107]. Moreover, anaplastic thyroid cancer cells and tumor tissues have a tendency to express CD133 specifically. Ge et al. designed an effective CD133-targeting AP-1 DNA aptamer. In vivo tumors formed from anaplastic thyroid cancer FRO cells seemed to particularly accumulate the shortened aptamer AP-1-M from its progenitor AP-1, which looked to have a greater binding affinity for CD133. In vitro and in vivo, the combination of shortened AP-1-M and doxorubicin significantly reduced CD133-positive FRO cell growth and promoted cell death [108].

The CD133 aptamer has also been used for targeted delivery of a glutaminolysis inhibitor to cancer stem cells. The GLS1 inhibitor telagenastat (CB-839) was loaded into PEGylated gold nanoparticles covalently conjugated to the CD133 aptamer. It was shown that the resulting complex significantly reduced the viability of CD133-positive brain cancer cells in a dose-dependent manner compared to the individual components of the assembled nanopreparation [109].

CD133 aptamers were used for targeted delivery of paclitaxel to CD133+ lung cancer stem cells. For this, the aptamer was conjugated with PLGA-PEG nanoparticles loaded with paclitaxel. When compared to the components alone, the resulting complex demonstrated significantly increased targeting and cytotoxicity of lung cancer stem cells in vitro and in mice that had reduced tumor mass [110]. CD133 aptamers have also been used for successful targeted delivery of gefitinib to lung cancer stem cells. CD133 aptamer was conjugated with DSPE-PEG2000 nanomicelles loaded with gefitinib [111]. In another study, a CD133 aptamer and dextran-coated mesoporous silica nanoparticles were used to target doxorubicin to tumor cells in CD133+ (HT29) colorectal cancer cells. The resulting complex significantly increased cellular uptake and had increased cytotoxicity on HT29 (CD133+) cells [112]. The CD133 aptamer has also been used to deliver DTX-loaded liposomes. The resulting complex significantly reduced lung cancer cell proliferation and improved therapeutic efficacy, had very good tumor targeting ability, and exhibited significant antitumor activity in A549 tumor mice with very low systemic toxicity [113].

#### 2.4.9. EGFR Aptamer

Epidermal growth factor receptor (EGFR) is a transmembrane glycoprotein of 170 kD and receptor tyrosine kinase (RTK) from the ERB-B family [114]. A variety of human malignancies, including breast, glioma, and lung cancers, have been linked to EGFR [115].

Many aptamers have been developed for EGFR. In cell culture, it has been demonstrated that EGFR aptamers interact with both monomeric and heterodimeric versions of EGFR as well as with other tyrosine kinase receptors. Tyrosine kinase receptors are known to be overexpressed in highly aggressive tumor cells. Anti-EGFR aptamers prevent the activation of downstream transcription factors and tumor growth and then induce apoptosis in tumor cells that are not affected by existing therapies [116].

The E07 RNA aptamer, CL4 RNA aptamer, and TuTu22 DNA aptamer are the most prevalent nucleic acid-based EGFR aptamers [60]. For instance, it has been demonstrated that the E07 RNA aptamer may bind EGFR with nanomolar affinity [117].

The unaltered RNA aptamers were chosen against the purified extracellular domain of EGFR. These aptamers were used for gold nanoparticle delivery to tumor cells or to capture EGFR-expressing glioma cells [115].

Aptamer R13 was discovered by screening cancer cells that overexpressed EGFR. Photodynamic treatment was performed using the R13 aptamer. Trimalonic acid was used to modify C70 fullerene, which was then conjugated with the R13 aptamer. Even after being conjugated to TF70, the R13 aptamer maintained good binding properties, and the TF70-R13 conjugate’s photodynamic treatment activity was significantly higher than that of TF70 alone [118].

Recently, conjugates of EGFR aptamer and an antibody against epidermal growth factor 2 receptor (ErbB2), as well as a conjugate of EGFR and an anti-PD-L1 antibody, have been developed. These new aptamer-antibody conjugates combine their inhibitory properties to increase the cancer cell killing activity while maintaining the targeting capacity of both parental moieties. Moreover, the anti-EGFR aptamer’s conjugation with the immunomodulatory antibody enabled effective T-cell activation and redirection against cancer cells, boosting the cytotoxicity compared to two initial agents [119].

Anti-EGFR aptamers were conjugated to various types of nanoparticles. Lv and colleagues constructed EGFR aptamer-conjugated polymer nanoparticles carrying a short hairpin RNA-expressing plasmid to repress survivin, which is overexpressed in non-small cell lung cancer. Using the obtained NPs, efficient gene transfection and drug delivery to tumor cells were observed. For EGFR-positive non-small cell lung cancer cells treated with these nanoparticles, inhibition of proliferation, induction of apoptosis, suppression of expression of the target survivin protein, and increased drug cytotoxicity were observed compared to cells treated with unmodified NP [120].

To improve the efficacy of cisplatin against cervical cancer cells, Chen et al. created cisplatin-loaded albumin nanoparticles conjugated with an EGFR aptamer. At the same time, both free and conjugated aptamers were able to attach to EGFR-positive HeLa cervical cancer cells [121].

Interesting research comparing the effectiveness of anti-EGFR aptamers with cetuximab was carried out by Kang et al. Two lipid nanoparticles (NPs) were created in this work, loaded with paclitaxel and quantum dots, and then coupled with cetuximab or EGFR aptamers. Both NPs entered the tumor more effectively than nontargeted NPs and significantly reduced tumor development [122].

Dong et al. also created a gene delivery system in EGFR-positive tumors. In this complex, the EGFR aptamer was conjugated to a liposome loaded with siRNA against the SATB1 protein. This delivery system’s ability to specifically target choriocarcinoma cells, drastically lower SATB1 expression, induce tumor growth inhibition, and further cause the death of EGFR-overexpressing choriocarcinoma cells has been demonstrated in cell lines. Importantly, SATB1 expression was suppressed by this gene delivery system in mouse choriocarcinoma xenografts with a tumor weight inhibition rate of 81.4% [123].

#### 2.4.10. HER2 Aptamer

Human epidermal growth factor receptor 2 (HER2, also known as ErbB2) is a protein that regulates cell proliferation and differentiation via a signaling cascade. HER2 protein overexpression is seen in 15%–20% of all breast cancers, as well as stomach, ovarian, and lung malignancies [124]. HER2 overexpression has also been linked to an increased risk of brain metastases, resistance to some chemotherapy medications, and cancer recurrence more often [125].

Many HER2-targeted delivery systems have been developed employing synthetic ligands, including aptamers, affibodies, peptides, and antibodies and their fragments [126,127]. Additionally, several HER-specific aptamers have been developed [115,126,128,129]. Typically, they exhibit excellent specificity and affinity for their targets [128,129]. Kim et al. created an anti-HER2 RNA aptamer that can be used for imaging HER2-positive cancers [127]. Kazem et al. also developed a DNA aptamer library for HER2-positive cells [126]. Interestingly, siRNAs directed against B-cell lymphoma have also been delivered using HER2-specific aptamers [130]. Mahlknecht et al. created a trimeric HER2 aptamer (named 2-2(t)) that had improved binding to HER2 compared to the monomeric version and had a higher antiproliferative capacity when tested in vitro on gastric cancer cells overexpressing HER2 [131].

To accomplish HER2-targeted breast cancer treatment, a HER2-targeted aptamer combined with the chemotherapy medication DM1 was also developed. The internalization of this complex into the HER2-overexpressing SKBR3 and BT474 cancer cell lines was successfully demonstrated by the authors. Moreover, in vivo research using BT474 xenografted mouse models showed that DM1 could be delivered to the tumor tissue precisely thanks to aptamer recognition [132].

#### 2.4.11. VEGF Aptamer

Vascular endothelial growth factor (VEGF) is an important regulator of tissue vascular development. VEGF has four different isoforms in mammals, one of which (VEGF165) is a marker related to tumor growth and metastasis [133,134]. Owing to the therapeutic significance of VEGF, several VEGF aptamers have been created [135]. The best-known VEGF aptamer is pegaptanib, which has been approved by the FDA for the treatment of age-related macular degeneration [135].

Some VEGF aptamers have been developed for delivery drug systems in cancer. Recently, Xie et al. synthesized pegaptanib-TDNs and investigated their antitumor and antiangiogenic effects. HUVEC and Cal27 cell proliferation was reduced by pegaptanib-TDNs, showing dependency on concentration. It was shown that loading pegaptanib onto TDNs boosted its antagonistic effects on VEGF. The authors hypothesized that pegaptanib-TDNs may circulate in the body for a long time and withstand different types of enzymatic degradation. These elements may improve the in vivo antiangiogenic and antitumor effectiveness of pegaptanib [136].

Fu and colleagues developed a multifunctional nanoparticle that included a VEGF aptamer and cytosine DNA fragments grafted onto the surface of superparamagnetic iron oxide NPs (SPION). When loading this complex with daunomycin and TMPyP, strong chemotherapeutic and phototherapeutic effects on cancer cells were observed. This effect occurred due to damage to mitochondrial membranes by reactive oxygen species, which eventually triggered apoptosis of tumor cells [137].

#### 2.4.12. EpCAM Aptamer

The transmembrane glycoprotein CD326, also known as EpCAM, is overexpressed in various solid tumor types [138]. In 97.7% of colon cancers, 90.7% of stomach cancers, 87.2% of prostate cancers, and 63.9% of lung malignancies, high levels of EpCAM expression were observed [139]. EpCAM is considered a cancer stem cell marker. It regulates the proto-oncogenes c-myc, e-fabp, cyclins A and E and activates the Wnt signaling pathway [140]. Shigdar et al. first presented an EpCAM RNA aptamer with a binding affinity of approximately 55 nM, which was successfully internalized by cells with the EpCAM receptor [141].

Many investigations have been conducted on the development of complexes using the EpCAM aptamer. In vitro experiments showed that aptamer-conjugated NPs were taken up more readily by tumor cells expressing EpCAM than unconjugated NPs in EpCAM-expressing cell lines [142].

A bispecific EpCAM-CD44 aptamer was developed by fusing two single aptamers with a dsRNA adapter. This aptamer conjugate effectively binds CD44 and EpCAM simultaneously and demonstrates a tumor-suppressing effect in mouse xenograft models. EpCAM-CD44 bispecific aptamers have a significantly improved circulating half-life compared with CD44 alone or aptamer EpCAM, are well tolerated by the host, and do not elicit innate immune responses [143].

Liet et al. developed a complex of EpCAM aptamer-conjugated PLGA nanoparticles encapsulated with curcumin. Since free curcumin was toxic to both HT29 cells and nontarget HEK293T cells, this complex was significantly more effective against EpCAM-expressing HT29 cells [142].

Xiang et al. demonstrated that 2–3 DOX molecules are intercalated into the EpCAM aptamer [144]. For targeted delivery of DOX to colon cancer cells, complexes were constructed in which the EpCAM aptamer was conjugated with DOX-loaded mesoporous silica nanoparticles. The results of this study showed that cellular uptake and cytotoxicity in SW620 cells were significantly higher in the complex where the aptamer was present, and this complex significantly inhibited EpCAM expression on tumor cells [142].

There are a number of drugs conjugated to the EpCAM aptamer. For example, Yoon et al. have used nucleoside analogs (gemcitabine, 5-FU) or cytotoxic agents (monomethylauristatin E or maytansine derivative 1) and the EpCAM aptamer targeting pancreatic cancer. All four of these drugs were successfully conjugated with the EpCAM aptamer, which could specifically deliver them to cancer cells without affecting normal cells. These drug conjugates with the EpCAM aptamer demonstrated significant inhibition of tumor cell growth, while they had no effect on normal cells [145].

The EpCAM aptamer was used as an active targeting system for the delivery of doxorubicin and survivin siRNA to breast cancer stem cells. As a result of the fusion of the aptamer and siRNA, chemoresistance was overcome, self-renewal of cancer stem cells was inhibited, and the survival rate of mice with tumors was increased [146].

In one investigation, the EpCAM antibody was directly compared to the EpCAM aptamer in vivo, and the aptamer was shown to be superior to the antibody [138].

#### 2.4.13. Spiegelmers

Spiegelmers are target-binding oligonucleotides that are synthesized from nonnatural L-nucleotides. Spiegelmers, like aptamers, fold into different forms to bind targets with high affinity and selectivity. L-RNA-based Spiegelmers have the benefit of being less vulnerable to acidic pH than DNA-based oligonucleotides [147]. It has been shown in animal models that Spiegelmers can be effective in various areas of medicine [148].

A PEGylated L-oligoribonucleotide called olaptized pegol (NOX-A12) can bind to the CXCL12 chemokine involved in the life cycle of chronic lymphocytic leukemia cells. NOX-A12 neutralizes CXCL12, resulting in reduced protective activity of the bone marrow and lymph node microenvironment. Phase I/II clinical trials were conducted in the treatment of 28 patients with relapsed or refractory chronic lymphocytic leukemia with Spiegelmer NOX-A12 in combination with bendamustine and rituximab. The findings of Michael Steurer et al. show that the use of NOX-A12 has the desired pharmacodynamic effect due to the effective mobilization of chronic lymphocytic leukemia cells. The 86% response rate and >80% 3-year overall survival rate are better than those achieved by rituximab alone and in recent rituximab combination trials [149].

For better comparison, the main features of DNA nanostructures and their potential applications are summarized in Table 1.

## 3. Delivery of DNA-Based Nanomaterials

### 3.1. Biodistribution and Biosafety of DNA-Based Nanomaterials

As of right now, the majority of oligonucleotide treatments (and nearly all licensed nucleic acid preparations) concentrate on either local delivery (for example, to the eyes or spinal cord) or hepatic delivery. As the liver is a strongly perfused organ, absorption of bigger nanoparticles and free oligonucleotides can happen quickly before renal clearance. Moreover, the liver has very high concentrations of receptors that might facilitate fast absorption and/or recycling. The development of effective methods for extrahepatic systemic distribution remains a key objective in the field of oligonucleotide treatment, despite the fact that other organs, such as the kidneys and spleen, are also locations of oligonucleotide accumulation [228,229].

Oligonucleotides are hydrophilic, negatively charged polymers that do not pass the plasma membrane by themselves [230]. Cellular DNAses are thought to break oligonucleotides within cells. On the other hand, the degradation process is linked to the uptake pathway of DNA nanostructures [13]. In addition, there are off-target interactions, toxicity depending on the sequence and chemical composition of oligonucleotides, and saturation of endogenous RNA processing pathways [231].

Data on the biodistribution of unmodified therapeutic DNA nanostructures, including aptamers, are limited. All unmodified oligonucleotides (including aptamers) have serious pharmacokinetic problems, including metabolic instability and rapid renal filtration without nonspecific protein binding [232]. For example, the half-lives of unmodified nucleotide aptamers in the blood are on the order of 2 min. Thus, short aptamer stability is a serious issue.

Nucleic acid preparations must resist extracellular degradation [233], prevent the release of the drug bound to specific plasma proteins from circulation [234], and avoid removal by the reticuloendothelial system. Nucleic acid drug platforms must cross the capillary endothelium to the desired target, cross the plasma membrane, avoid lysosomal degradation [235], and be delivered to a specific target in the cell [230]. Therefore, the most commonly used strategies to improve drug delivery and safety from nucleic acids include chemical modification, covalent conjugation with cell-targeting or cell-penetrating molecules, and the use of nanoparticles to lessen adverse effects and boost the treatment efficiency of drug delivery technologies [230]. Most of the aptamers are within 50 nucleotides in length and fall within the renal filtration range. In the case of polymer conjugation, aptamers largely reduce renal filtration [236]. Additionally, chemical modifications are proposed to be incorporated into nucleotide sugars or internucleotide phosphodiester linkages to overcome this problem for aptamers and increase their serum half-life. In addition, mirror L-DNA can solve the problem of low stability of natural DNA in blood serum, which affects the pharmacokinetics and biological distribution [237].

To date, many DNA-based carriers have been shown to have little cytotoxicity and are mainly used as a hub for integrating drug loading, targeting, and release modules together [238]. There is currently very limited information on the safety and efficacy of treatments using DNA nanostructures. Some experiments have been carried out on cell culture, as well as several animal studies [239].

One of the biggest challenges for TDNs in further clinical use is whether TDNs can cause unpredictable gene recombination or deleterious effects in the liver or kidneys. Nevertheless, it was shown that TDN has better biological safety, lower biotoxicity, and higher transport efficiency than other DNA carriers in cell culture [240]. For example, the biosafety of TDN for DOX delivery has been shown [15].

Zhang et al. demonstrated that triangular origami DNA loaded with doxorubicin could be an effective and safe innovation platform for the treatment of breast cancer in nude mice [241]. Recently, the safety of DNA origami at physiological pH for noncancerous cells and its cytotoxicity for cancer cells at the pH of solid tumors have been demonstrated [242].

In general, aptamers have also been shown to have little or no side effects and are safe. Aptamers did not lead to the activation of the immune system [243]. Many analyses in preclinical and early clinical trials have not shown complement activation. Additionally, no off-target side effects were identified [244]. In 2022, the first phase I human clinical trial of the ApTOLL DNA aptamer was conducted and the results in healthy male volunteers demonstrated great safety and a suitable pharmacokinetic profile of aptamer. The infusion’s termination resulted in the highest concentration, and the mean half-life was 9.3 h. At any dose or with any researched form of administration, serious adverse effects or biochemical abnormalities were not seen. However, the study showed no accumulation of ApTOLL [55].

However, the shelf life of DNA nanostructures can raise safety concerns. For instance, because DNA sequences in nanostructures are complicated and unpredictable, foreign DNA may at random interact with cellular RNA and result in other possible chronic toxicities. Moreover, the immunological response to DNA nanostructures in vivo has not been well studied. Immunostimulatory activity through a TLR9-independent mechanism should not be disregarded, despite the fact that numerous research claim that CpG-free DNA nanostructures have little immunogenicity [245].

### 3.2. Cellular Uptake of DNA Nanostructures

Endocytosis is the process through which macromolecules, their complexes, and large particles enter the cell. Endocytosis is classified into two types: phagocytosis, which consumes particles larger than 250 nm, and pinocytosis, which consumes fluid and particles that phagocytosis does not consume. Pinocytosis occurs in all cell types via four different mechanisms: macropinocytosis, clathrin-mediated endocytosis, caveolar-mediated endocytosis, and clathrin- and caveolin-independent endocytosis [246]. Other pathways, with the exception of macropinocytosis, are directly controlled by cargo molecule activity. Cargo molecules bind to receptors and form receptor–ligand complexes attracting certain effectors to specific parts of the plasma membrane [247].

**Figure 6:**
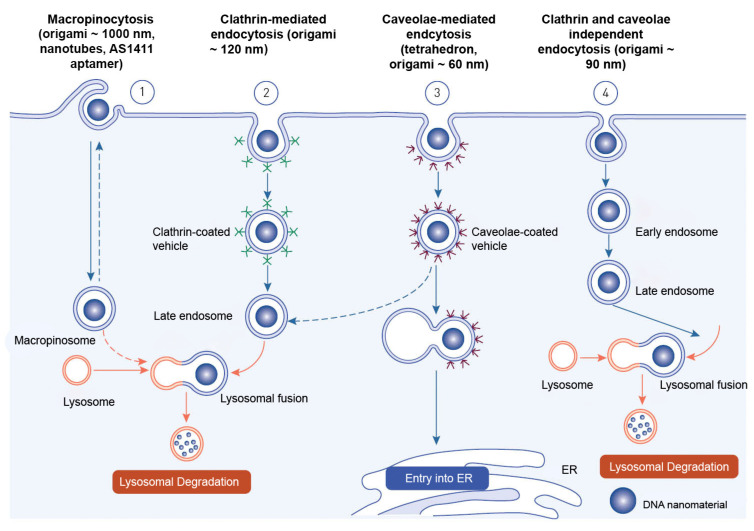
Endocytotic pathways involved in the internalization of DNA-based nanomaterials.

Depending on their size and the proteins involved, oligonucleotides enter cells through a variety of endocytosis processes (Figure 6). Despite the fact that all endocytosis routes lead to the creation of endosomes, molecules entering via distinct channels may end up at different downstream locations. The majority of absorbed oligonucleotides end up in late endosomes and lysosomes. There is, nevertheless, some partial translocation to other membrane-bound compartments. Oligonucleotides inside the endomembrane compartment are pharmacologically inactive, and only a tiny percentage of internalized oligonucleotides may reach the cytosol and nucleus on their own. The method of injection may influence oligonucleotide cellular uptake [248].

Receptor-mediated endocytosis and macropinocytosis are the two primary methods of internalization of DNA-based nanomaterials. The activity of cargo molecules and other proteins controls clathrin-mediated and caveola-mediated endocytosis [249]. Free oligonucleotides, oligonucleotide conjugates, or oligonucleotide-carrying nanocarriers are internalized by endocytosis once they reach the cell surface [248].

The mechanism of endocytosis of DNA-based nanomaterials is not fully understood. The endocytosis process has only been examined for a relatively small variety of DNA nanostructures. The structure, chemical makeup, and cell type of the target cells must all be taken into account in studies of endocytosis and cell fate of each unique design. DNA nanostructures that have been absorbed typically end up being trapped and degraded in lysosomes. As a result, methods that enhance endolysosomal escape and encourage targeted drug delivery to the cytosol and nucleus are needed. These issues will also be resolved by researching a nonendocytic route for DNA nanostructures [238].

Nanocarriers enter cells mostly through endocytosis and are sent to different organelles inside the cell [250]. The loaded drug is typically released from the nanocarriers either extracellularly, in the microenvironment, or intracellularly into the tumor, primarily through cellular uptake via endocytosis [251].

#### 3.2.1. Delivery Mechanism of DNA Tetrahedrons

It is known that the size of nanostructures has a significant impact on the type of endocytic route used. The TDNs are basically 7 to 20 nm in diameter [151]. By adjusting its orientation, TDN, in contrast to other DNA nanostructures, reduces electrostatic repulsive interactions and redistributes the TDNs’ uneven charge at the membrane surface, allowing it to actively penetrate the cell membrane using the vertex [252]. Tetrahedrons may be taken up through caveolin-dependent receptor-mediated endocytosis, according to a theory put out by Liang and colleagues. The receptor(s) in this uptake are not exactly established [5]. TDNs are carried into lysosomes by microtubules in a highly organized manner after entering a cell through membrane caveolin, preserving structural stability in the cell cytoplasm for up to 12 h [252]. While TDNs maintain their structural stability within cells for a relatively long time, their location in lysosomes prevented them from being used as efficient delivery systems. Liang et al. further modified TDN with signal peptides to direct TDN to certain organelles to prevent tetrahedra from being degraded in lysosomes. They discovered that TDNs are quickly internalized via a caveolin-dependent pathway [5].

Ma et al. showed that caveolin-1 and micropinocytosis-related protein sorting nexin 5 were involved in TDN endocytosis [8].

#### 3.2.2. Delivery Mechanism of DNA Origami

It has been demonstrated that DNA origami nanostructures’ larger size and stronger compactness enable more effective internalization than single strand DNA or less compact structures. The DNA origami nanostructures can internalize even without transfection. [197]. This allows greater loading capacity and more complicated payload patterning. Because ligand–receptor recognition and cell internalization are sensitive in this range, DNA origami may become more useful than smaller DNA nanocarriers. Some problems remain unsolved, for example, how stable DNA origami may be in a biological environment and how immunogenic exogenous nucleic acids might be [31].

The increased permeability and retention effect for DNA origami structures allow targeted tumor drug delivery since these structures passively accumulate in solid tumors. Furthermore, DNA nanostructures can pass through mouse or human skin, implying potential applications as transdermal drugs for melanomas. DNA origami structures were recently discovered to preferentially pile in the kidney of a mouse, indicating the possibility for kidney disease treatments [31]. It has been shown that some types of drug-containing origami are capable of efficiently locating and/or eliminating cancer cells, even when they are not specifically modified with targeting moieties [253].

DNA origami can enter the cell through a number of endosomal pathways, including macropinocytosis, scavenger receptor-mediated endocytosis, clathrin- and caveolar-mediated endocytosis, and clathrin- and caveolar-independent endocytosis. The location where the origami DNA contacts the cell surface determines the endosomal pathway [254].

DNA origami that have similar geometrical properties and molecular weights but differ in structural rigidity and packing density show varying levels of internalization. In the H1299 and DMS53 cell lines, investigation of four different DNA origami nanostructures of two different sizes and shapes on a flow cytometer indicated that large origami are internalized far more often than small origami [255]. Although having the same molecular weight, it was demonstrated that rod-shaped origami had a greater absorption efficiency than tetrahedral-shaped origami. More uptake efficiency is typical for larger origami because they can interface with the membrane and capture more receptors than smaller origami [145].

In mouse tumors, the passive accumulation of origami DNA in three different shapes, namely triangle, rectangle, and tube, was investigated using quantum dots [241]. After 24 h of injection, it was observed that more triangles than tubes accumulated at the tumor location. Which DNA origami nanostructure structural design is most effective for cellular internalization is still unknown [256].

The most logical way to achieve targeting for DNA origami is to include a ligand that can be recognized by its specific molecule in the cell type of interest. DNA aptamers are an example of such a ligand. Other cell-targeting approaches include the incorporation of folate, cell-penetrating peptides, and the use of immunoinert proteins [253].

The DNA origami must either exit the endosomal compartment it is imprisoned in after uptake or release its payload. Specific endosome-avoidance techniques have been developed for drug-loaded origami structures. In one instance, the anthracycline antibiotic daunorubicin was placed into a rod-shaped DNA origami structure. This strategy led to resistance bypass in MDR-1 cells. This DNA origami structure prevented it from disintegrating in the serum [257]. The ability of the DNA origami structure to maintain integrity for a sufficiently long time is the first condition for use as a drug carrier [253].

In several cell lysates (CP-A, End1/E6E7, MCF-10A, HeLa, and MDA-MB-231), Harris et al. showed that 2D and 3D DNA origami were stable for 12 h [258].

#### 3.2.3. Delivery Mechanism of DNA Nanotubes

Due to their pore-like characteristics, DNA nanotubes have the ability to alter the membrane potential. Alternatively, they might have cytotoxic effects after being absorbed by the cells and could perhaps obstruct endosomal system traffic. Several studies have shown that DNA nanotubes with high rigidity have a higher cellular uptake efficiency, especially for nanotubes, compared to other DNA structures [48].

To investigate the NT internalization mechanism, an endocytosis inhibition experiment was carried out in which the uptake of the single-stranded DNA nanotubes was assessed in GL261 cells in the presence of various endocytosis inhibitors. In the presence of DMA, cellular uptake of the DNA nanotubes was inhibited by 44% on average and by 57% in the presence of fucoidan. In addition, treatment with the LatB inhibitor reduced the internalization of single-stranded DNA nanotubes by 20%, suggesting that macropinocytosis is involved in the internalization of DNA nanotubes. The internalization of DNA nanotubes was not reduced by inhibitors of other endocytosis pathways or other uptake mechanisms [259].

To evaluate the uptake of DNA nanotubes by MCF-7 cells, Shen and colleagues utilized carbazole-based cyanine, which displays a substantial increase in fluorescence when linked to DNA helices [260]. Studies using confocal microscopy demonstrated that the intact DNA nanotubes were taken up by cells and gathered in lysosomes. Moreover, electroporation can transfer DNA nanostructures modified with spermidine into cells [261]. Similar to DNA origami, DNA nanotubes can be modified with ligands for efficient cellular uptake [256].

It has also been shown that DNA nanoribbons (a kind of DNA nanotube) may be internalized into the cytoplasm of H460 cells through clathrin and lipid raft-mediated endocytosis using different endocytosis inhibitors [262]. Additionally, after 2 h of incubation, these nanoribbons demonstrated endosomal escape capability [256].

Hamblin et al. showed enhanced uptake by HeLa cells of DNA nanotubes obtained by rolling circle amplification. Large negatively charged DNA nanotubes could penetrate into the cell even without conjugation with transfection agents, as the authors suggest, due to the thick shell of DNA chains in the structures. However, it is still unknown how DNA nanotubes are taken up by cells [263].

#### 3.2.4. Aptamer Delivery Mechanism

Aptamers are approximately 20 times smaller in size than antibodies [264]. Their small size may help them penetrate tumor tissues more effectively [265]. For the in vivo use of aptamers in targeted delivery or as therapeutic candidates, the kind of cellular uptake is crucial. Aptamers are characterized by two mechanisms for cellular uptake: receptor-mediated endocytosis and macropinocytosis [247].

When the aptamer interacts with the receptor in clathrin-dependent endocytosis, clathrin-coated pits are formed, and then clathrin-coated vesicle budding occurs. The clathrin coat detaches from the membrane and is broken down. Internalized aptamers end up in endosomes and lysosomes before being redistributed to other organelles based on the type of host cell. However, it is not yet known how exactly aptamers are distributed inside cells. A caveolin-dependent mechanism has been shown for the A10 aptamer conjugated with drug-loaded nanoparticles.

Receptors are also involved in the caveolar-mediated endocytosis of aptamers. Following aptamer-receptor interactions, it entails the production of caveolae vesicles, which is mediated by dynamin. Membrane proteins known as caveolins and cavins are found in membrane invaginations known as caveolae. Aptamers may move over the membrane to the caveolae invagination after binding to their target surface receptor. This endocytic pathway has been found for aptamer sgc8–drug conjugates with 5-fluorouracil [249].

Using the macropinocytosis inhibitor amiloride, another type of uptake of aptamers by target cells was established [266]. Macropinocytosis is controlled by the protein actin and is characteristic of nucleolin aptamers. The AS1411 aptamer is one of the most well-studied examples of cellular absorption. AS1411 endocytosis can occur in a variety of ways. Although macropinocytosis is the most widespread mechanism of AS1411 endocytosis, both clathrin- and caveol-dependent and caveol-independent pathways are implicated in AS1411 uptake [267]. Reyes-Reyes et al. discovered that nonmalignant skin fibroblasts and cancer cells had different mechanisms for uptake AS1411 (Hs27). The majority of cancer cells are susceptible to AS1411, including prostate cancer cells (DU145), whereas Hs27 cells are resistant to AS1411 [268]. Macropinocytosis is the predominant mechanism for internalization of AS1411 into cancer cells. Kotula and colleagues showed that AS1411 is not internalized by receptor-mediated endocytosis, which is the most common uptake mechanism for other aptamers [269].

Aptamer–drug conjugates have been reported to enter cells by receptor-mediated endocytosis. The medication is released from the aptamer in the lysosome and cleared away, allowing it to travel to the nucleus. When a medication is absorbed in an unspecific manner, drug efflux pumps can rapidly uptake pharmaceuticals out of the cell, limiting their concentration within the cell. This is the same mechanism that allows antibody–drug conjugates to deliver a larger concentration of medication than nonspecific cytotoxic medicines [138].

An aptamer compound created to penetrate into breast and prostate malignant tumor cells based on common receptor overexpression provides a good example of effective aptamer uptake and drug delivery. Three aptamers were present in this complex: apMNK2F, which specifically inhibits MAP kinase; IDA, which targets the 64 integrin receptor; and AS1411, which targets nucleolin. With the aid of AS1411 and IDA aptamers, the aptameric nanocomplex was internalized, enabling effective cell uptake, avoiding endosomal degradation, and nucleolin-mediated transport to the nucleus of target cells [270].

Many aptamers that are not internalized have cytotoxic activity through receptor binding and subsequent effects on various signaling pathways in the cell. However, this effect is mediated by signaling blockades and not by internal intracellular mechanisms. [271].

## 4. Conclusions and Future Perspectives

DNA is a molecular genetic information carrier that may be turned into useful nanomaterials in biomedicine and engineering [272]. Such properties of DNA, such as Watson–Crick base pairing, local rigid structures, a single system for recording hereditary information, structural features of functional groups, the possibility of modification with enzymes, and synthesis technologies, have led to the creation of numerous DNA-based nanomaterials [13]. Because of their structural diversity and scientific value, DNA-based nanomaterials have become one of the most appealing nanocarriers. DNA has excellent biocompatibility as a basic component of living organisms [8]. DNA strands hybridize with each other and can then be easily engineered into a functional nanostructure with high spatial programmability, such as designed DNA nanodevices compatible with the immune system and DNA-based drug-delivery vehicles (Table 2) [33].

DNA-based nanomaterials as nanocarriers have received much attention. In contrast to nanoparticles, DNA-based nanomaterials are more attractive due to the double helix, which has a robust predetermined 3D geometry that allows the prediction and design of the structures of more complex nucleic acid complexes. The assembly of DNA scaffolds through the presence of multiple binding sites provides an empty interior space for drugs. Due to electrostatic forces, positively charged molecules may be integrated into DNA nanoparticles owing to the polyanionic structure of DNA. It has recently been shown that synthesized DNA nanostructures can easily pass through the membrane barrier, unlike natural nucleic acids [412].

Moreover, DNA is used more effectively in diagnostics and cancer therapy by utilizing a range of nanomaterials, including nanosheets, liposomes, fluorescent, gold and magnetic nanoparticles, with a decreased danger of being severely damaged by intracellular nucleases. Chemotherapeutic drugs, photosensitizers, CpG oligonucleotides, siRNA, CpG, Cas9, and antibodies are just a few of the drugs or cargo molecules that researchers have thus far been able to deliver into target cells using DNA nanostructures.

Using targeted drug delivery systems built from DNA nanostructures functionalized by particular groups could address an issue with chemotherapy. Several chemotherapeutic medicines can form covalent and noncovalent bonds with DNA. For instance, DOX can successfully intercalate into GC-rich double-stranded DNA. Furthermore, functional nucleic acid aptamers can be included in DNA nanomaterial complexes to improve targeted specificity and greatly reduce unfavorable side effects. Due to the effective loading of photosensitizers, such as BMEPC and methylene blue, DNA nanocarriers have been commonly utilized in photodynamic therapy [413].

The development of DNA-based drug delivery systems for in vivo use is supported by evidence for the enhanced selectivity and effectiveness of DNA materials in vitro. It has been shown that origami and tetrahedra may carry medications into cancer cells without the need for transfection agents. Several studies on the delivery of chemotherapeutic agents have been shown to be effective in mouse models [238]. DNA-based nanocarriers containing the chemotherapy drug doxorubicin may be able to cross the barrier, significantly slowing the in vivo growth of U87MG tumors. It has also been found that DNA-based materials facilitate the delivery of large cargoes, including biomacromolecules and nanoparticles.

As DNA nanostructures are often internalized into antigen-presenting cells via the endocytic pathway, delivering synthetic oligonucleotides using DNA nanostructures is an effective approach for immunotherapy [238]. The use of DNA-based nanomaterials to trigger a specific type of T-cell immune response by delivering antigens through either exogenous or endogenous antigen processing and presentation pathways is one promising area of immunomodulation research [46]. DNA-based nanomaterials have also been investigated as therapeutic nucleic acid delivery systems for genes, antisense RNAs, and small interfering RNAs [414]. A simple method to create a delivery system based on a DNA nanostructure comprising antisense nucleotides and doxorubicin was introduced by Pan et al. [415]. To carry doxorubicin and two distinct antisense oligonucleotides to drug-resistant cancer cells for improved treatment, a multifunctional DNA origami-based nanocarrier was created.

The use of DNA-based nanomaterials in cancer clinical research is still in its early stages. Key information about potential clinical applications needs to be clarified. It is also necessary to study and elucidate the pharmacokinetics of DNA nanocarriers in vivo: their uptake, the use of targeted molecules, escape from degradation in lysosomes, passive accumulation, half-life, and clearance mechanism [416].

## Figures and Tables

**Table 1:** Characteristics of DNA tetrahedrons, DNA origami, DNA nanotubes, and aptamers and their applications.

DNA Nanostructure	Size	Structure	Synthesis Method and Assembly	Applications for Cancer Therapy
DNA tetrahedron	7 nm [150]– 20 nm [151].	Tetrahedral shape, double-boundle TDN [152].	Single-step synthesis method: TDN synthesized by mixing four single-stranded DNAs in one pot after a quick thermal annealing process; self-assembly [153]. Two-step synthesis method: self-assembly of TDN with fluorophore and amido bond formation with drug [154]. Isothermal synthesis of a DNA tetrahedron: 3D wireframe TDN synthesized by ring forming reactions in the presence of an initiator [155].	Drug delivery carriers: small molecules—doxorubicin [19], paclitaxel [156]; nucleic acid drugs: CpG [157], ASOs [158], siRNA [159], aptamers [150], RNase A [160]. Photodynamic therapy: carrier for iridium catalyst [25], therapy circulating tumor cells [161]. Cancer theranostics: [162]. Cancer cell detection and in vivo imaging: [150,163,164,165,166,167,168].
DNA origami	2D DNA origami ~100 nm [28], 3D origami ~ size up to 1000 nm [169].	2D DNA origami (rectangles, triangles, five-pointed star, etc.) [28], 3D DNA origami: tube [35], honeycomb [170], square [171] lattices, origami cage structure [172], 3D prism structures [173], cube [174], DNA nanoribbons [175].	Scaffolded self-assembly of DNA strands: single-stranded scaffold and over 200 short oligonucleotide “staple” strands are mixed and self-assembled in a single step in desired shapes [28]. Single-stranded tile assembly: single strands of DNA containing four domains associate into staggered duplexes, resulting in DNA lattices [176]. Simulated annealing algorithm for automatic generation of DNA origami: computes unique designs by utilizing shape annealing, by integration of shape grammars and the simulated annealing algorithm [32].	Immune engineering: encapsulates antibodies to antigen protein expressed on the surface of leukemia cells [174]. Drug delivery carriers: doxorubicin [35], p53 gene and doxorubicin [177], daunorubicin [37], MUC-1 aptamer and RNase A [178], cisplatin [179]. Photodynamic therapy: Au nanorods [180,181], Au NPs [175], BMEPC [38]. Cancer imaging and therapy: [182]. Biosensors: [183,184,185,186].
DNA nanotube	9 nm [187]–29.5 µm [188]	Tubular [189,190,191], triangular and square-shaped DNA nanotubes [43], L-, T-, and Y-shaped [192].	Single-stranded DNA tiles: ssDNA containing several domains hybridized with each other to form tiles [42,191]. Multi-crossover DNA tiles: Hybridization of DNA tiles and wrapping by intrinsic or external factors [193,194]. «Scaffolded» DNA Origami: The formation of nanotubes from DNA origami occurs via either direct self-assembly [171]. Multi-rungs: nanotubes are formed from single-stranded and cyclic DNA templates, when rigid vertices helped the cyclic DNA to create “rungs” and then assembled using perpendicular linkers [43].	Drug delivery carriers: doxorubicin [195], doxorubicin or paclitaxel with MUC-1 aptamer [196], CpG [197], cytochrome *c* [48], aptamer and ricin A [49], AuNPs [198,199], enzyme-responsive DNA nanotubes with targeting DOX release [200]. Cancer Diagnosis and Targeted Therapy: DNA nanotube–peptide biocomplex [196], anti-EGFR targeting with a pH-responsive controlled release of TW-37 [201]. Fluorescent Dyes: Cy3 [47], Blue-Red-Green [202]. Biosensors: detection of multiplexed biomarkers—microRNA-21 (miRNA-21) and glutathione (GSH) [203].
Aptamers	EpCAM aptamer ~2.09 nm [204]–PLA-MUC1aptamer nanoparticle 200 nm [205].	single-stranded DNA or RNA molecule; unique due to their secondary and tertiary structure [206]	Automated solid-phase synthesis [207], enzymatic synthesis (for evolutionary selection of modified aptamers) [208,209]. SELEX is process for generation aptamers, including initial library design, target preparation, PCR optimization, and single strand DNA separation [210]. Types of SELEX: Cell SELEX, Complex–Target SELEX, Genomic SELEX, Microfluidic SELEX (M-SELEX), Magnetic Bead-Based Microfluidic SELEX, Capillary Electrophoretic (CE) SELEX, Sol–Gel Method, AFM-SELEX, Toggle-SELEX [211].	Aptamer and aptamer–drug conjugates for targeted therapy: AS1411 (clinical study) [63]; chemotherapy, gene therapy, immunotherapy, radiotherapy, phototherapy [212]. Targeted real-time imaging and diagnostics: aptamer-based protein recognition [213], detection of cancer cells [214], detection of MUC-1 positive cells [215], capture and detection of circulating tumor cells [216,217], in vivo imaging tumors [218,219], prostate cancer detection [217], probe for contrast-enhanced in vivo cancer imaging [220], image-guided cancer thermotherapy [221], cancer-targeting therangostics probe [222], monitor DNA degradation [223], positron emission tomography [224], magnetic resonance imaging [225], PET imaging [226], CT imaging and therapy of cancer [82], PET/CT tumor visualization [227].

**Table 2:** DNA-based nanomaterials as drug delivery platforms for increasing the effect of drugs in tumors.

DNA-Based Nanomaterial	Method and/or Strategy for Delivering Drug	Target	Cell Line/Cancer	Payload	Reference
DNA tetrahedron	aptamer HER2 was anchored on tetrahedral framework nucleic acid via covalent bond	HER2	SKBR-3, MCF7, MCF-10A	HApt- tetrahedral DNA	[273]
DNA tetrahedron	TDNs conjugated with pegaptanib	VEGF	HUVECs, Cal27	Pegatinib	[136]
DNA tetrahedron	miR-21 or miR-155 or AS1411 modified DNA tetrahedron/metal–organic framework conjugates	nucleolin	MDA-MB-23, HepG2	Doxorubicin or camptothecin or rhodamine 6G or fluorescein	[274]
DNA tetrahedron	Methylene blue loaded into TDN via intercalation	cell apoptosis	B6F10, SCC7, MDA-MB231	Methylene Blue, PDT	[23]
DNA tetrahedron	PTX loaded into TDN via intercalation	MDR cell apoptosis	A549, PTX-resistant A549/T	Paclitaxel	[18]
DNA tetrahedron	ASOs loaded into DNA tetrahedrons conjugated with NLS peptide	c-raf	A549	ASO	[26]
DNA tetrahedron	One of the strands mirror TDN was biotinylated and conjugated to streptavidin. Various biotinylated enzymes can be loaded onto the streptavidin subunit of the hybrid.	apoptosis, gene modification, and carbohydrate hydrolysis	HeLa, Nano fibroblast cells	Caspase 3; Cre recombinase; β-galactosidase	[24]
DNA tetrahedron	Cy5, 5-FU, and AS1411 were conjugated with TDN	nucleolin	MCF10A, MCF7	5-Fluorouracil	[275]
DNA tetrahedron	AS1411 was linked with TDN	nucleolin	A549	AS1411	[276]
double-bundle DNA tetrahedron	56MESS was loaded into TDN through intercalation	EGFR	A431, A549, A549/DDP, MCF-7, and A2780	platinum-based DNA intercalator, 56MESS	[152]
DNA tetrahedron	TDN modified with MUC1 and AS1411aptamer; DOX-loaded via intercalation	MUC1, nucleolin	MCF7, HL-7702	Doxorubicin	[277]
FUdR-DNA-affibody chimera	10 FUdR molecules were attached to 5′ end of each DNA strand	HER2	BT474, MCF-7	5-Fluorodeoxyuridine	[27]
DNA mini-hexahedron (DMH)	hibridization of AS1411 sequence with one strand of DMH	nucleolin	A549	miRNA-1246 and miRNA-21	[278]
DNA tetrahedron	DOX and TMPyP4 were loaded via intercalation into AS1411-modified TDN	nucleolin	HeLa	DOX and TMPyP4	[279]
DNA origami	intercalation	cell apoptosis	MDA-MB-231, MDA-MB-468, MCF-7	Doxorubicin	[195]
DNA origami	RNase A-DNA origami conjugate	MUC1	MCF-7	MUC1-modified RNase A	[178]
DNA origami	intercalation	cell apoptosis	MCF-7	BMEPC (3,6-bis [2-(1-methylpyridinium) ethynyl]-9-pentyl-carbazole diiodide), PDT	[38]
DNA origami	intercalation	MDR cell apoptosis	HL-60/ADR	Daunorubicin	[37]
DNA nanotubes	cholesterol-modified DNA nanotubes conjugated with cytochrome *c*	Cell apoptosis	HeLa	Cytochrome C	[48]
self-assembled DNA nanotubes	Cy3-labeled and folate-conjugated nanotubes	FR (folate receptor)	Nasopharyngeal epidermal carcinoma KB cells	Cy3	[47]
DNA nanotube	DOX and PTX were loaded into the modified with MUC-1 capsulated DNA nanotube–peptide	MUC-1, integrin receptors (αv β3)	MCF-7	Doxorubicin or paclitaxel	[196]
AS1411 aptamer	conjugation of aptamer AS1411 with indocyanine green (ICG) or with acridine orange ligand C8	nucleolin	B16	C8—acridine orange derivative or ICG	[280]
AS1411	AS1411-conjugated pluronic F127 mixed micelles encapsulating doxorubicin	nucleolin	MCF-7	Doxorubicin	[281]
AS1411	AS1411 was conjugated with self-assembled HSA-paclitaxel nanoparticle (HSA—Human serum albumin)	nucleolin	MCF-7, MCF-10A and 3T3	Paclitaxel	[282]
AS1411	Aminated AS1411 covalently modify graphene oxide-chitosan oligosaccharide-γ-polyglutamic acid nanocarrier loaded with doxorubicin	nucleolin	Beas-2B, HeLa	Doxorubicin	[283]
AS1411	AS1411 aptamer-functionalized PEG-PLA micelle were constructed through an emulsion/solvent evaporation strategy for the codelivery of doxorubicin and miR-519c	nucleolin	HepG2	Doxorubicin and miR-519c	[284]
AS1411	AS1411 attached onto the surface chitosan-coated MSNs	nucleolin	C26, MCF-7, 4T1, CHO	Doxorubicin and antimiR-21	[285]
AS1411	AS1411 is covalently conjugated to the PAMAM dendrimer loaded with camptothecin	nucleolin	A549	Camptothecin	[286]
AS1411	AS1411-PGG-PTX conjugate (PGG: L-γ-glutamyl-glutamine)	nucleolin	U87 MG, and HUVEC	Paclitaxel	[287]
AS1411	Aptamer/hyaluronic acid-bifunctionalized microemulsion	Nucleolin, CD44	U87, HUVEC	Docetaxel + Shikonin	[288]
AS1411	AS1411 conjugated with PEGylated PAMAM loaded with camptothecin	nucleolin	HT29, C26, CHO	Camptothecin	[289]
AS1411	AS1411-conjugated DC-Chol/DOPE liposomes (ASLP). The ASLP/siRNA complex was formed through electrostatic interaction between ASLP and siRNA	nucleolin	A375, MDA-MB-231, A549, C6, HeLa, A375 tumor xenograft mice	anti-BRAF siRNA	[290]
AS1411	Aptamer was covalently bound to Au nanoparticles	nucleolin	HeLa, NHDF and HEC-1-A	C8 or Imiquimod	[291]
AS1411	AS1411 was conjugated with PAMAM-PEG encapsulated wth 5-fluorouracil	nucleolin	MKN45	5-Fluorouracil	[292]
AS1411	AS1411, influenza hemagglutinin peptide and clofarabine were conjugated into self-assembled peptide nanoparticles with siRNA and doxorubicin	nucleolin	MCF-7, L0-2	Doxorubicin, siRNA(TK1), clofarabine	[293]
ATP-AS1411 bivalent aptamer	ATP-AS1411 was conjugated to DOX-loaded Si nanoparticles	ATP and nucleolin	CHO	Doxorubicin	[294]
AS1411	Camptothecin was encapsulated in β-CD-(PCL-PAEMA)_21_ micelle modified with AS1411 aptamer	nucleolin	MCF-7, 4T1, L929	Camptothecin	[295]
AS1411	siRNA with conjugate AS1411-photo-sensitive oligonucleotide (OliP)	nucleolin	4T1	siRNA, irridation with 365 nm light	[296]
AS1411	AS1411-conjugated PLGA-lecithin-PEG nanoparticles loaded with paclitaxel and Nile red	nucleolin	MCF-7, GI-1, L929, HMEC	Paclitaxel + Nile red	[297]
AS1411	Doxorubicin-loaded AS1411-conjugated nanoparticles	nucleolin	MCF-7	Doxorubicin	[298]
AS1411	AS1411-conjugated HPAEG polimer could form self-assembed nanoparticles loaded with doxorubicin	nucleolin	MCF-7	Doxorubicin	[299]
AS1411	AS1411 and siRNA attached to the surfaces of MSNs nanoparticles loaded doxorubicin	nucleolin	MDA-MB-231	Doxorubicin + siRNA(TIE2)	[300]
AS1411	AS1411 was conjugate with DOPE-sphingomyelin-cholesterol-DSPE-PEG2000 liposome loaded with paclitaxel and siRNA	nucleolin	MCF-7	Paclitaxel + siRNA	[301]
AS1411	AS1411 was conjugated with extracellular vesicles loaded with microRNA precursor let-7 or VEGF siRNA	nucleolin	MDA-MB-231	miRNA let-7 or siRNA (VEGF)	[302]
AS1411	Electrostatic interaction between fluorescent gold nanocluster-conjugated chitosan and AS1411	nucleolin	A549	Methotrexate	[303]
AS1411	AS1411-capped FRET-based two-photon MSNs loaded with doxorubicin	nucleolin	MCF-7, HEK293	Doxorubicin	[304]
AS1411	AS1411-PAMAM-PEG-fluorescent tags modified magnetic zinc-doped iron oxide nanoparicle with the loading doxorubicin and siRNAs	nucleolin	MDA-MB-231, 4T1, MCF-10A	Doxorubicin + siRNA HSP70 or HSP90. NIR/MR	[305]
AS1411	Silica nano supra-assembly nanoparicle with conjugating DOX, cytochrome c and AS1411	nucleolin	HCT116	Doxorubicin	[306]
AS1411	AS1411-conjugated PAMAM grafted persistent luminescence nanoparticles loaded with doxorubicin	nucleolin	HeLa, 3T3	Doxorubicin	[307]
AS1411	AS1411- absorptio, dsDNA and MMP-2 cleavable peptide-fabricated gold nanocage vehicle which could load doxorubicin and siRNAs	nucleolin	NCI-H889	Doxorubicin and siRNAs	[308]
AS1411	AS1411 decorated via the absorption of a single-stranded thymidine (T)-rich tail of oligonucleotide T20 onto the surface ZnO-gated porphyrinic metal–organic framework loaded with doxorubicin	nucleolin	HeLa, NIH3T3	Doxorubicin, PDT	[309]
AS1411	AS1411 was anchored onto the surface of the MnO2-coated and loaded with acriflavine and HMME liposomes	nucleolin	SKOV3, HL-7702	Acriflavine, HMME. Sonodynamic therapy (SDT)	[310]
AS1411	AS1411 aptamer-based three-way junction pocket DNA nanostructure loaded with doxorubicin	nucleolin	PC-3, 4T1, CHO	Doxorubicin	[311]
AS1411	The protoporphyrin IX loading AS1411r-conjugated upconversion nanoparticle	nucleolin	MCF-7, HeLa	Protoporphyrin IX, PDT, NIR	[312]
AS1411	As1411-conjugated HSA nanoparticle loaded with 5- Fluorouracil and BpT (2-benzoylpyridine thiosemicarbazide copper II)	nucleolin	Bel-7402	5-Fluorouracil, BpT	[313]
AS1411	As1411 was conjugated with chitosan nanoparticles loaded with erlotinib	nucleolin	A549	Erlotinib	[314]
AS1411	As1411 was conjugated with polydopamine-functionalized CA-(PCL-ran-PLA) nanoparticles loaded with docetaxel	nucleolin	MCF-7	Docetaxel	[315]
AS1411	As1411 was conjugated with PEG-PLA-DPPC lipopolymersome loaded with camptothecin	nucleolin	HT29, C26	Camptothecin	[316]
AS1411	Doxorubicin inserted in the aptamer AS1411 was encapsulated in the aqueous interior of liposome	nucleolin	MCF-7/Adr	Doxorubicin	[317]
AS1411	As1411 was conjugated with Mn-doped mesoporous silica nanoparticles containing 5-aza-2-deoxycytidine and docetaxel	nucleolin	MCF-7	5-aza-2-deoxycytidine + docetaxel	[318]
AS1411	AS1411-conjugated albumin nanoparticles loaded with docetaxel	nucleolin	CT26	Docetaxel	[319]
AS1411	TMPyP4 and doxorubicin were then physically attached to the AS1411-conjugated Au NPsAu nanoparticles	nucleolin	HeLa, Dox-resistant MCF-7R	TMPyP4 + Doxorubicin, PDT	[320]
AS1411	AS1411-conjugated chimeric self-assembled polymersomes loaded with SN38	nucleolin	HT29, CHO	SN38 (7-ethyl-10-hydroxycamptothecin)	[321]
AS1411	AS1411 immobilized on the surface DNA-modified gold nanoparticles loaded with doxorubicin	nucleolin	HeLa, NIH-3T3	Doxorubicin	[322]
AS1411	AS1411-conjugated gold nanoparticles loaded with doxorubicin or AZD8055	nucleolin	MCF-7, OMM1.3, Mel202	Doxorubicin or AZD8055	[323]
AS1411	AS1411 was introduced into zeolitic imidazolate framework-8 nanoparticles loaded with doxorubicin	nucleolin	HeLa, HEK 293T	Doxorubicin	[324]
AS1411	AS1411 was immobilizing over composite [γ-cyclodextrin-based metal–organic framework embedded with graphene quantum dots and modified with PEGMA]. Doxorubicin was encapsulated within this composite	nucleolin	MCF-7, L929	Doxorubicin	[325]
AS1411	AS1411-conjugated PAMAM-10C-10C-PEG nanoparticles loaded with bcl-xl shRNA (10C—10-bromodecanoic acid)	nucleolin	A549, L929	bcl-xl shRNA	[326]
AS1411	AS1411 and PEG were conjugated with molybdenum disulfide nanosheets loaded with doxorubicin and coated with polydopamine (PDA) layer	nucleolin	MCF-7	Doxorubicin, NIR	[327]
AS1411	AS1411 aptamer-tethered DNA nanotrains loaded with anthracycline drugs	nucleolin	HeLa, L02	Doxorubicin or, epirubicin, or daunorubicin	[328]
AS1411-ATP fusion aptamer	Polyplexes by doxorubicin -loaded AS1411-ATP fusion aptamer and PEG-pDQA	nucleolin	MCF-7/DOX	Doxorubicin	[329]
AS1411	AS1411 was conjugated onto cell membrane capsules loaded with doxorubicin	nucleolin	QGY-7703, Hepli	Doxorubicin	[330]
AS1411	AS1411-PEG was conjugated with PDA-coated CA-PLGA nanoparticles loaded with docetaxel	nucleolin	MCF-7	Docetaxel	[331]
AS1411	AS1411 and TGN peptide modified PEG-PCL nanoparticles loaded with docetaxel	nucleolin	C6, bEnd.3	Docetaxel	[332]
Cy5.5-AS1411	Covalent assembly of Cy5.5-AS1411 aptamer conjugate on the surface of graphene oxide wrapped doxorubicin-loaded mesoporous silica nanoparticles	nucleolin	MCF-7	Doxorubicin	[333]
AS1411	AS1411 aptamer was conjugated with PEG-PDLLA micelle loaded with triptolide	nucleolin	MIA PaCa-2	Triptolide	[334]
AS1411	Click-nucleic-acid containing platform for codelivery of rapamycin, anti-PFKFB4 siRNA and aptamer AS1411	PFKFB4, nucleolin	HEK 293, T1-luci	Rapamycin and anti-PFKFB4 siRNA	[335]
AS1411	AS1411-conjugated PLGA-PVP nanoparticles loaded with doxorubicin	nucleolin	A549	Doxorubicin	[336]
AS1411	AS1411-coniugated star-shaped glucose-core PCL-PEG nanoparticles containing LNA-anti-miR-214	Nucleolin, miR-214	A2780	anti-miR-214	[337]
AS1411	DNCA lipid-AS1411 nanoparticles. AS1411 interact via Watson–Crick and p-stacking with DNCA	nucleolin	A549, MCF-7, K562	AS1411	[338]
AS1411 and S2.2 aptamers	AS1411 and S2.2 aptamers were conjugated to the surface of liposomes coated with gold nanoshells and loaded with docetaxel	Nucleolin, MUC1	MCF-7	Docetaxel	[339]
AS1411	AS1411 was covalently attached to the surface of the dextran-coated PLA-PEI nanoparticles which co-loaded with camptothecin and survivin-shRNA	nucleolin	C26, CHO	Camptothecin, survivin-shRNA	[340]
AS1411-polydopamine	AS1411 was conjugated wth polydopamine-modified M-PLGA–TPGS nanoparticles loaded with docetaxel	nucleolin	HeLa	Docetaxel	[341]
AS1411	AS1411 and a BHQ2-labeled ATP aptamers incorporated into a hybrid micellar nanoparticle	nucleolin, ATP	HeLa	BHQ2, PDT	[342]
AS1411	AS1411 and cytochrome *c* were conjugated with mesoporous silica nanoparticles loaded with doxorubicin	nucleolin	HepG2	Doxorubicin	[343]
AS1411	AS1411 was conjugated with magnetic PLGA-PEG nanospheres loaded with doxorubicin	nucleolin	C6, L929	Doxorubicin	[344]
AS1411	AS1411 and cRGD were conjugated with gold nanocluster, functionalized with NIR dye; doxorubicin was linked with gold nanoconjugate	nucleolin	U87MG, MCF-7, L02, A549	Doxorubicin, NIR	[345]
AS1411	Dual-functional probe composed of gold nanoparticles, catalytic Zn2+-dependent DNAzyme, doxorubicin, targeted AS1411 aptamer and acid-decomposable ZnO quantum dots. The Zn2+-dependent ligation DNAzyme and AS1411 aptamer were assembled onto the gold nanoparticles (GNPs) via Au-S bonding.	nucleolin	HeLa	Doxorubicin and miRNA-21	[346]
Apta 12	DOX-APTA12 conjugate	nucleolin	MCF-10A MDA-MB-231	Doxorubicin + gemcitabine	[347]
MUC1 aptamer	MUC1 aptamer–siR-29b chimera was sinthesized	MUC-1, PTEN	OVCAR-3	miRNA-29b	[348]
MUC1 aptamer	MUC-1 aptamer was conjugated InP/ZnS quantum dots/nanohydrogel fluorescent composite loaded with paclitaxel and sodium oxamate	MUC-1	MCF-7	Paclitaxel + sodium oxamate	[349]
MUC1 aptamer	MUC-1-responsive DNA motif with hairpin structure (smart sensor) was conjugated with doxorubicin-loaded MSNs	MUC-1	MCF-7, Hs578bst	Doxorubicin	[350]
MUC1 aptamer	MUC1 aptamer-peptide anti-HSP70 peptides (P8 and P17) conjugates	MUC-1	MCF-7/ADR, A549, HepG2	Doxorubicin	[351]
MUC1 aptamer (aptA)	1,10-phenanthroline can be intercalated within aptA when complexed with Fe(II) ions	MUC-1	MCF-7	1,10-phenanthroline	[352]
MUC1 aptamer	Doxorubicin-incorporated multivalent aptamer–siRNA conjugate	MUC-1	MCF-7	BCL2-specific siRNA + doxorubicin	[353]
MUC1 aptamer	MUC1-dimer aptamer-calcium carbonate complexes loaded with epirubicin and melittin	MUC-1	MCF7, C26, HepG2	Epirubicin and melittin	[354]
MUC1 aptamer	MUC1aptamer was conjugared with ferritin nanoparticles loaded with epirubicin	MUC-1	colon cancer cells	Epirubicin	[355]
MUC1 aptamer	Conjugate of KLA–MUC1 aptamer loaded with doxorubicin to the surface DNA nanoparticles	MUC-1	MCF-7	Doxorubicin and KLA peptide	[356]
MUC1, AS1411 and ATP aptamers	Aptamers-dendrimer conjugate	MUC-1, nucleolin, ATP	MCF-7, C26, CHO	Epirubicin	[357]
MUC1 aptamer and AS1411	MUC1 aptamer incorporated heparin and AS1411 incorporated heparin are decorated on the surface of protamine sulfate/CaCO3 nanoparticle loaded with CRISPR–Cas9 plasmid	MUC-1, nucleolin	HeLa, MCF-7, HEK 293T	CRISPR–Cas9 plasmid (for FAK knockout)	[358]
MUC1 aptamer	MUC1 aptamer was conjugated to surface PLA-PEG nanoparticles loaded with doxorubicin	MUC-1	A-549	Doxorubicin	[359]
MUC1 aptamer	Doxorubicin-conjugated MUC-1 aptamer-armed PEGylated SPIONs	MUC-1	MDA-MB-231	Doxorubicin	[360]
MUC1 aptamer	Gold coated Fe2O3 nanoparticles conjugated with MUC-1 aptamer	MUC-1	MCF-7, CHO	Photothermal therapy	[361]
MUC1 aptamer	MUC1 aptamer was conjugated with genistein-miRNA-29b-loaded hybrid nanoparticles	MUC-1	A549	Genistein and miRNA-29b	[362]
MUC1 aptamer	MUC1 aptamer-conjugated chitosan nanoparticles loaded with siRNA and docetaxel	MUC-1	SKBR3	IGF-1R siRNA and docetaxel	[363]
MUC1 aptamer	ZnSe/ZnS quantum dot-Protoporphyrin IX-MUC1 aptamer conjugate	MUC-1	HeLa, RAW	Protoporphyrin IX, PDT	[364]
MUC1 aptamer	MSNs loaded with safranin O or with doxorubicin and conjuugated with MUC1 aptamer	MUC-1	MDA-MB-231, MCF-10-A	Safranin O or doxorubicin	[365]
MUC1 aptamer	MUC1 aptamer was conjugated to chitosan-SN38 nanoparticles	MUC-1	HT-29, CHO	SN38	[366]
MUC1 aptamer	MUC1 was conjugated with chitosan-coated albumin nanoparticles loaded with paclitaxel	MUC-1	MCF7, T47D	Paclitaxel	[68]
MUC1 aptamer	Clofarabine, ara-guanosine, gemcitabine, and floxuridine to replace all natural nucleosides in aptamer sequences	MUC-1		Therapeutic Nucleoside Analogues	[367]
MUC1 aptamer	Aptamer-conjugated liposome containing docetaxel	Tubulin, MUC-1	MCF 7, MCF 10A, MDA-MB-231, HeLa, Hep G2	Docetaxel	[368]
MUC1 aptamer	Doxorubicin was intercalated into self-assembled CpG-MUC1-hydrogel	MUC-1	MCF-7, A549, HepG-2, RAW264.7	Doxorubicin	[369]
MUC1 aptamer	MUC1 aptamer was conjugated with liposomes (sDPPC, cholesterol, PEG 2000-DSPE and Mal-PEG 2000-DSPE) encapsulated gold nanocages and doxorubicin	MUC-1	MCF-7	Doxorubicin, gold nanocages + NIR light irradiation	[370]
MUC1 aptamer	MUC1 aptamer-conjugated chitosan nanoparticles loaded with docetaxel and siRNA	MUC-1	SKBR3, CHO	Docetaxel and cMET siRNA	[371]
5TR1-aptamer	5TR1 aptamer conjugated to surface PEGylated liposome loading doxorubicin	MUC-1	C26	Doxorubicin	[372]
5TR1-aptamer	Doxorubicin was loaded on the modified 5TR1-GC (5TR1 to add a GC loop)	MUC-1	MDA-MB-231, MCF-10A	Doxorubicin	[373]
5TR1-aptamer	5TR1 aptamer was conjugated with chitosan-modified PLGA nanoparticles	MUC-1	MCF7, C26	Epirubicin	[374]
5TR1 aptamer	SPION-5TR1 aptamer conjugate loading epirubicin	MUC-1	C26, CHO-K1	Epirubicin	[375]
PSMA aptamer	PSMA aptamer-anchored PLGA-b-PEG-COOH nanoparticles loaded with docetaxel	PSMA	LNCaP	Docetaxel	[78]
PSMA RNA aptamer	PSMA aptamer-conjugated liposome loaded with doxorubicin	PSMA	LNCaP	Doxorubicin	[79]
PSMA aptamer (A10)	A10 was conjugated with DSPE-PEG2000 liposome-CRISPR/Cas9 chimeras	PSMA, PLK-1	LNCaP, PC-3	CRISPR/Cas9 gRNA target PLK1	[376]
A10 aptamer	A10-3.2 aptamer was conjugated with FoxM1 siRNA-loaded cationic nanobubbles	PSMA	LNCaP	FoxM1 siRNA	[377]
PSMA aptamer	PSMA aptamer was conjugated with doxorubicin-loaded H40-PLA-PEG micelles	PSMA	CWR22Rν1	Doxorubicin	[80]
A10 aptamer	A10 aptamer-functionalized doxorubicin-polylactide conjugate	PSMA	LNCaP, PC-3	Doxorubicin	[378]
A10-3.2 aptamer	A10-3.2 aptamer-modified ultrasound-responsive nanodroplets loading siRNA	PSMA, CAT-1	22RV1, PC-3, 16HBE	siRNA against siCAT-1 transporter	[379]
MRP1-CD28 aptamer	MRP1-CD28 aptamer conjugate	MRP1, CD28	B16F10, chemotherapy-resistant tumors	Translational whole-cell vaccine (Aptvax) has been developed based on this MRP1-CD28 aptamer conjugate	[86]
CD28 aptamer	CAR-like multivalent aptamer nanoparticles (X-polymers): murine CD28Apt7 + the tetramer of CTLA-4 + RNA aptamer (Del60) + folic acid labeled ssDNA	CD28, CTLA-4	mouse melanoma B16 cell	CAR-T-cell immunotherapy	[87]
CD38 aptamer	CD38 aptamer-doxorubicin conjugate	CD38	MM cells, CD38-negative cells, HDLM2, Jeko1, L428, K299	Doxorubicin	[380]
CD44 aptamer	CD44 aptamer was conjugated to the surface of PEGylated liposomes	CD44	A549, MDA-MB-231, the CD44(-) cell line, NIH/3T3	-	[381]
CD44 aptamer	CD44 aptamer-doxorubicin conjugate	CD44	Breast cancer cells	Doxorubicin	[382]
CD44 aptamer	CD44 aptamer was conjugated with PEGylated liposomes loaded with siRNA	CD44	MDA-MB-231-Luc2-GFP	siRNA	[383]
CD44 thioaptamer	CD44 thioaptamer was conjugated with PAMAM-PEG/miRNA-145 nanoparticles	CD44	MDA-MB-231, MCF-7	miRNA-145	[384]
CD44-anti-PD-L1 aptamer	Liposomes have been loaded with loaded DOX and IDO1 siRNA and conjugated to CD44 and anti-PD-L1 DNA aptamers	CD44, PD-1/PD-L1 interaction	MDA-MB-231 cells; mouse: 4T1 cells	IDO1 siRNA, Doxorubicin	[93]
Anti-EGFR-CD44 aptamer	An anti-EGFR-CD44 aptamer was conjugated with solid lipid dexamethasone-modified nanoparticles modified loaded with doxorubicin.	EGFR, CD44	MDA-MB-468 cell	Doxorubicin	[94]
CD44 DNA aptamer	Five-Part Pentameric nanocomplex incorporating CD44 DNA aptamer for cellular targeting and thiolated hyaluronic acid to stabilize Au NPs	CD44	HeLa, SKOV-3, C13, NIH3T3, SH-SY5Y	Doxorubicin	[95]
CD71- aptamer	CD71 aptamer was conjuugated with XQ-2d-MMAE	CD71	OCM-1	Monomethyl auristatin E (MMAE)	[98]
CD117 aptamer	CD117 aptamer-methotrexate conjugates	CD117	CD117-expressing HEL cells	Methotrexate	[101]
B19 aptamer	B19 aptamer-conjugated PAMAM G4C12 dendrimer nanoparticles loaded with paclitaxel and temozolomide	CD133	U-87, C6	Paclitaxel + temozolomide	[385]
CD133 aptamer	CD133 aptamer-PLGA-PEG micelle loaded with paclitaxel	CD133	A549	Paclitaxel	[110]
CD133 aptamer	PEGylated CD133aptamer-doxorubicin conjugate	CD133	Hep3B, Huh7, HEK293T	Doxorubicin	[107]
CD133 aptamer	CD133 was conjugated covalently to the self-assembled PEGylated carboxymethylcellulose-SN38-conjugate nanoparticles	CD133	HT29, CHO	SN38	[386]
Thiolated CD133 aptamer A15	A15 aptamers were conjugated with curcumin-loaded liposomes (EPC, CHOL and DSPE-PEG2000-MAL)	CD133	DU145	Curcumin	[387]
CD133 aptamer	CD133 aptamers were conjugated propranolol-loaded poly(lactic-co-glycolic acid) nanoparticle	CD133	HemSC	Propranolol	[388]
CD133 aptamer	CD133 aptamer was conjugated with salinomycin-loaded PEGylated nanoparticles	CD133	Saos-2, U-2 OS, MG-63	Salinomycin	[389]
CD133 aptamer	Gefitinib-loaded nanomicelles conjugated with CD133 aptamers	CD133	A549, A431	Gefitinib	[111]
CD133 aptamer (A15) + EGFR (CL4)	Salinomycin-loaded poly(lactic-co-glycolic acid) nanoparticles A15 or CL4-conjugted or nontargeted salinomycin-loaded nanoparticles.	CD133, EGFR	Huh7, Hep3B	Salinomycin	[390]
CD133 aptamer	CD133 aptamer was conjugated with polymer-micellar NPs composed of poly(styrene-b-ethylene oxide) (PS-b-PEO) and PLGA and labeling with ^89^Zr	CD133	Human GBM cancer stem cells	Temozolomide, idasanutlin	[391]
CD133 aptamer	Conjugating CD133 aptamers to DTX liposomes (DSPE-PEG2000-CHOL-SPC)	CD133	A549	Docetaxel	[113]
CD133 aptamer	All-trans retinoic acid (ATRA)-loaded lipid-polymer nanoparticles conjugated with CD133 aptamers	CD133	Saos-2, U-2 OS	ATRA	[392]
CD133 aptamer	CD133 aptamer shortened form (AP-1-M) was conjugated with doxorubicin	CD133	FRO cells	Doxorubicin	[108]
Cs5 aptamer	Doxorubicin was loaded into the Cs5 aptamer to form a chimera.	CD133	HCT116	Doxorubicin	[393]
CD133 aptamer + angiopep-2 (An2)	Angiopep-2 and CD133 RNA aptamers were conjugated on exosomes as vehicles loaded with temozolomide and O6-benzylguanine	CD133, An2	U87MG, GSC	Temozolomide + O6-benzylguanine	[394]
CD133 aptamer	Propranolol-loaded CD133 aptamers conjugated liposomes-in-microspheres	CD133	HemSCs	Propranolol	[395]
HER2 aptamer	HER2 aptamer was conjugated with mertansine	HER2	BT-474, MDA-MB-231	Mertansine	[396]
HER2-EGFR aptamer	Bivalent HER2 aptamer-EGFR siRNA chimera	HER2, EGFR	BT474, SKBR3, MDA-MB-231, MCF7, Hs578 T	siRNA	[397]
HER2 aptamer	HER2 aptamer-functionalized pH-sensitive β-cyclodextrin -capped doxorubicin-loaded MSNs	HER2	SKBR3, MCF7, HEK-293T	Doxorubicin	[398]
HER2 aptamer	MMAE-conjugated HER2 oligobody (the cotinine (cot)-body, cot-linker, aptamer and MMAE)	HER2	SKBR3, NCI-N87	MMAE	[399]
HER2 aptamer	pH-responsive micelle-like nanoparticles carrying a Texas red-fluorescently labeled HER2 aptamer	HER2	SKBR3, MCF7, HeLa	HER2-aptamer	[400]
HER2 aptamer	HER2 was conjugated to the surface curcumin-loaded HSA nanoparticles	HER2	SKBR3	Curcumin	[401]
HER2 aptamer	HER2 aptamer/Ursolic acid + doxorubicin nanoparticles constructed as carrier-free nanodrugs	HER2	BT474	Ursolic acid + doxorubicin	[402]
Aptamer A6	Aptamer-labeled liposomal nanoparticle using different saturated (HSPC and DPPC) and unsaturated (POPC and DOPC) lipids	HER2	SKBR3, MCF7, MDA-MB-231	Doxorubicin	[403]
Aptamer A6	Aptamer-labeled P-gp siRNA encapsulated PLGA or PLGA-PEG nanoparticles	HER2, P-gp	SKBR-3, 4T1-R, MDA MB-231, MCF-7	Aptamer-labeled P-gp siRNA	[404]
HER2 aptamer and AS1411 aptamer	Dual aptamer-decorated DNA hydrogel	HER2 + nucleolin	SKBR-3, MCF7, MDA-MB-23	Doxorubicin	[405]
HER2 aptamer	HER2 densely grafted on gold nanostars	HER2	SKBR-3, MCF-10A	HApt-AuNS	[406]
HER2 aptamer	Carboxylated chitosan-coated (pH-responsive), doxorubicin-loaded aptamer- MSN bioconjugates	HER2, EGFR	SKBR-3, MCF7	Doxorubicin	[407]
VEGF aptamer	DNM and TMPyP were physically assembled with aptamer of VEGF + cytosine (C)-rich DNA fragment (gc-34)	VEGF	MCF-7	Daunomycin + TMPyP, PDT	[61]
EpCAM RNA aptamer	RNA aptamer-conjugated PEGylated liposome loaded with doxorubicin	EpCAM	CHO-K1, C26	Doxorubicin	[408]
EpCAM	EpCAM aptamer-survivin siRNA fusion, combined with doxorubicin treatment doxorubicin-resistant subline of the MCF-7 cells	EpCAM	MCF-7	Doxorubicin and survivin siRNA	[146]
FOXM1 aptamer	FOXM1 aptamer conjugated with DOX-CPT-loaded HA-b-PCL nanopolymersomes	FOXM1	A549, SK-MES-1	Doxorubicin, camptothecin	[409]
Sgc8c aptamer	Aptamer sgc8c–doxorubicin conjugate	PTK7	NB-4, Ramos	Doxorubicin	[410]
Aptamer BAFF-R	BAFF-R aptamer–siRNA conjugate	BAFF-R	Jeko-1, Z138	siRNA	[411]
